# The Mechanism of Gene Targeting in Human Somatic Cells

**DOI:** 10.1371/journal.pgen.1004251

**Published:** 2014-04-03

**Authors:** Yinan Kan, Brian Ruis, Sherry Lin, Eric A. Hendrickson

**Affiliations:** Department of Biochemistry, Molecular Biology, and Biophysics, University of Minnesota Medical School, Minneapolis, Minnesota, United States of America; Duke University, United States of America

## Abstract

Gene targeting in human somatic cells is of importance because it can be used to either delineate the loss-of-function phenotype of a gene or correct a mutated gene back to wild-type. Both of these outcomes require a form of DNA double-strand break (DSB) repair known as homologous recombination (HR). The mechanism of HR leading to gene targeting, however, is not well understood in human cells. Here, we demonstrate that a two-end, ends-out HR intermediate is valid for human gene targeting. Furthermore, the resolution step of this intermediate occurs via the classic DSB repair model of HR while synthesis-dependent strand annealing and Holliday Junction dissolution are, at best, minor pathways. Moreover, and in contrast to other systems, the positions of Holliday Junction resolution are evenly distributed along the homology arms of the targeting vector. Most unexpectedly, we demonstrate that when a meganuclease is used to introduce a chromosomal DSB to augment gene targeting, the mechanism of gene targeting is inverted to an ends-in process. Finally, we demonstrate that the anti-recombination activity of mismatch repair is a significant impediment to gene targeting. These observations significantly advance our understanding of HR and gene targeting in human cells.

## Author Summary

Gene targeting is important for basic research and clinical applications. In the laboratory, gene targeting is used to knockout genes so that loss-of-function phenotypes can be assessed. In the clinic, gene targeting is the gold standard to which most gene therapy approaches aspire. One of the most promising tools for gene targeting in humans is recombinant adeno-associated virus (rAAV). The mechanism by which rAAV performs gene targeting has, however, remained obscure. Here, we surprisingly demonstrate that the normally single-stranded rAAV performs gene targeting via double-stranded intermediates, which are mechanistically indistinguishable from standard plasmid-mediated gene targeting. Moreover, we establish the double-strand break (DSB) repair model as the paradigm to describe human gene targeting, and delineate the dynamics of crossovers in this model. Most unexpectedly, we demonstrate that when a meganuclease is used to introduce a chromosomal DSB to augment gene targeting, the mechanism of gene targeting is inverted such that the chromosome becomes the “attacker” instead of the “attackee”. Finally, we confirm that the anti-recombination activity of mismatch repair is a significant impediment to gene targeting. These observations advance our understanding of the mechanism of human gene targeting and should readily lend themselves to developing improvements to existing methodologies.

## Introduction

Gene targeting is the process of intentionally altering a genetic locus in a living cell [1]. This technology has at least two applications of significant importance. One application is the clinically-relevant process of gene therapy, which in a strict sense, involves correcting a preexisting mutated allele of a gene back to wild-type (a “knock-in”) to alleviate the pathological phenotype associated with the mutation. The second application is the inactivation of genes (“knockouts”), a process in which the two wild-type alleles of a gene are disrupted to determine the loss-of-function phenotype associated with that particular gene. Importantly, although these two processes are conceptually reciprocal opposites of each other, they are mechanistically identical because both require a form of DNA double-strand break (DSB) repair (DSBR) termed homologous recombination (HR).

During HR, as elaborated predominately in yeast [2], the ends of the invading double-stranded DNA (dsDNA) are resected to yield 3′-single-stranded DNA (ssDNA) overhangs [3], which, in turn, are substrates for Rad51. Rad51 is a strand exchange protein [4], which facilitates the base pairing of the invading strand with its homologous chromosomal donor. After second strand capture, a recombination intermediate is generated with two Holliday Junctions (HJs) that is identical to the intermediate of plasmid-based gene targeting that has been well-defined in yeast [2], [5]–[7]. Resolution of this intermediate requires different combinations of polymerases, helicases, nucleases and ligases that result in distinct recombination products. Importantly, human cells express all of the HR genes needed to carry out gene targeting [1]. However, because of the robust competing pathway of DSBR known as non-homologous end joining [8], gene targeting events occur rarely in mammals [9]–[11]. Indeed, despite valiant efforts — in particular by the Baker laboratory [10], [12], [13] — the low targeting efficiency of plasmid-based dsDNA vectors has prohibited a systematic characterization of recombination intermediates in mammalian cells. To gain better insight into the mechanism of human gene targeting it is crucial to establish a more vigorous gene targeting system.

Russell and coworkers have demonstrated that recombinant adeno-associated virus (rAAV) can target the human genome with frequencies up to 1% {[14]; Figure S1}, which is 3 to 4 orders of magnitude higher than plasmid-mediated gene targeting. rAAV has subsequently become a powerful tool to engineer knockout and knock-in mutations in the human genome [1], [15]. Despite its utility, the mechanism of rAAV integration remains elusive although it is clear that the recombinant virus, which encodes no viral proteins, must utilize host DSB pathways for its integration. Interestingly, since only single-stranded genomes can be packaged into virions (Figure S1), many reviews [16]–[18] have postulated that rAAV gene targeting is mediated by single-strand assimilation.

Here we systematically analyzed the molecular features of gene targeting intermediates. In contrast to popular belief, we demonstrate that rAAV gene targeting is mediated predominantly by the DSBR model of HR [19] with double-stranded viral DNA utilized as a substrate. Specifically, we analyzed the retention of single nucleotide polymorphisms (SNPs) — markers that allowed us to distinguish donor from recipient DNA — during gene targeting and random integration. We show, in contrast to lower eukaryotes and murine embryonic stem cells [20]–[23] that the positions of HJ resolution are evenly distributed along the homology arms of the targeting vector (Figure S2) in two independent human cell lines. In addition, we demonstrate that rAAV gene targeting events are mechanistically distinguishable from random integration events. Most unexpectedly, we observed that in the presence of chromosomal DSBs rAAV switches to a chromosome-initiated, ends-in recombination mode (Figure S3), which greatly augments the gene targeting process. A detailed analysis of the intermediates of the ends-in recombination reaction revealed that HJ resolution is preferred over synthesis dependent strand annealing (SDSA) or HJ dissolution in DSB-induced gene targeting when conversion of a large selection marker is required. Finally, we demonstrate that one of the largest hindrances to human gene targeting is the anti-recombination activity of mismatch repair. These observations greatly expand our understanding gene targeting and its underlying HR mechanism in human cells.

## Results

### The HPRT targeting system

**Figure 1:**
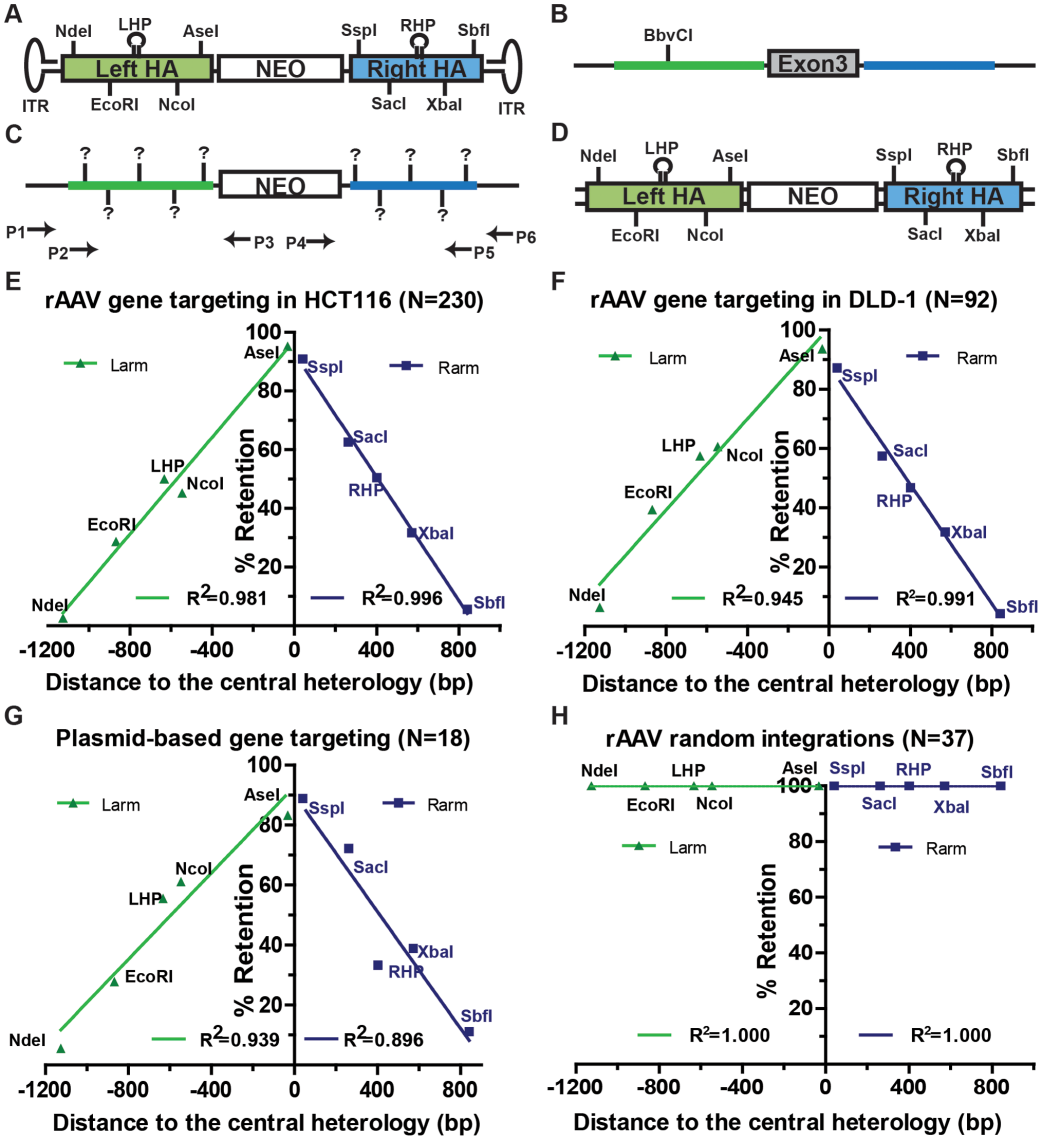
Gene targeting is marked by a characteristic SNP retention signature. (A) The rAAV targeting vector. The NEO cassette (white rectangle) is flanked by the homology armss (green and blue rectangles). NdeI, EcoRI, NcoI, AseI, SspI, SacI, XbaI and SbfI represent vector-specific restriction sites created by SNPs. LHP/RHP represent vector-specific palindromes (lollipops) created by introducing SNPs. The flanking hairpins represent inverted terminal repeats. (B and C) The HPRT locus before and after gene targeting. The NEO cassette replaces exon 3 (grey) of HPRT upon gene targeting. The theoretical positions of the viral markers are indicated in bold vertical lines and (?) symbols. The arrows represent PCR primers. P1xP3 and P4xP6 amplify the left and right homology arms of the gene targeted clones, respectively, and P2xP3 and P4xP5 amplify the homology arms of the randomly integrated clones, respectively. The LHP destroys a chromosomal BbvCI restriction site upon integration. (D) The dsDNA targeting vector. All symbols are defined above. (E, F, G and H) SNP retention signatures of rAAV gene targeting for HCT116 and DLD-1 cell lines, plasmid dsDNA gene targeting and random integration, respectively. The distance to the central heterology (cartooned as a vertical black line) is calculated from the inner ends of the homology arms. Markers on the left homology arms are indicated with negative distances. Green and purple lines represent the linear regression between the retention frequency and the distance of the viral markers for the left and right homology arms, respectively.

The X-linked *hypoxanthine phosphoribosyltransferase* (*HPRT*) locus is widely used as a negative selection marker [14], [24]. Inactivation of HPRT by a single round of gene targeting confers 6-thioguanine resistance in male cells. In our system, a rAAV targeting vector (Figure 1A) was assembled to disrupt exon 3 of HPRT (Figure 1B) with a neomycin (NEO) drug-resistance cassette. Following G418 selection, gene targeting and random integration events could be distinguished based on their 6-thioguanine resistance or sensitivity. In order to differentiate the viral DNA from its chromosomal counterpart, each homology arm of the virus was marked with 4 SNPs that generated unique restriction enzyme recognition sites. In addition, a 22 bp hairpin structure, which is refractory to the mismatch repair machinery [12], [25] that was generated by the inclusion of 3 to 4 SNPs, was also introduced into each homology arm (Figure 1A). The homology arms of the targeted and randomly integrated clones could be amplified from the integrated loci (Figure 1C) using diagnostic PCRs. Primer pairs P1xP3 and P4xP6 (gene targeting primers) specifically amplified the left and right homology arms of targeted clones, whereas P2xP3 and P4xP5 (random integration primers) amplified the randomly integrated clones with intact homology arms (Figure 1C). The retention of the viral SNPs and hairpins was analyzed either by restriction enzyme sensitivity or DNA sequencing, or both.

### Gene targeting is characterized by a linear gradient loss of the homology arms

To elucidate the molecular mechanism of rAAV gene targeting, it was important to characterize which parts of the homology arms were integrated into the genome. Since the retention of SNPs can be influenced by mismatch repair, gene targeting was initially performed in the mismatch repair-deficient, male HCT116 and DLD-1 cell lines, which are deficient in MLH1/MSH3 and MSH6, respectively [26], [27]. In the later part of this paper we demonstrate that while the mismatch repair status of a cell affects the frequency of gene targeting it importantly does not affect the SNP retention profile. After rAAV infection, cells were selected with G418 and 6-thioguanine. A total of 230 (for HCT116) and 92 (for DLD-1) correctly targeted clones were confirmed by PCR and analyzed for the retention frequency of viral SNPs, which was then plotted against the position of the SNPs on the homology arms (Figure 1E and F and Tables S1 and S6). Strikingly, the viral SNPs were retained in a virtually linear gradient pattern: R^2^ equaled 0.981 and 0.996 for the left and right homology arms, respectively, in HCT116 cells (Figure 1E) and 0.945 and 0.991 for the left and right homology arms, respectively, in DLD-1 cells (Figure 1F). The inner SNPs had the highest chance of retention, whereas the outer markers were mostly lost during gene targeting. The linear SNP retention profile suggested that the positions of HJ resolution were evenly distributed throughout the homology arms because when HJ resolution occurs, the viral homology arms distal to that position will not be retained. Importantly, the linear retention profile observed in human cells for gene targeting contrasts with the exponential SNP retention reported for meiotic recombination in yeast and *Drosophila* and for mitotic recombination in yeast and mouse embryonic stem cells {[7], [20]–[23]; Figure S2}, which implied that the dynamics of HJ formation/resolution during gene targeting in human somatic cells may be different from similar processes in other organisms.

To determine if the even distribution of HJ resolution was intrinsic to rAAV-mediated gene targeting or was a general feature of gene targeting in human cells, a parallel transfection experiment was performed using a plasmid-based vector that was identical to rAAV except that it was double-stranded and it did not contain the inverted terminal repeats (Figure 1D). Ultimately, 18 correctly targeted clones were recovered despite the extremely low targeting efficiency of this approach. SNP analysis revealed an indistinguishable linear retention curve (Figure 1G and Table S2). Thus, the even distribution of HJ resolution is a general characteristic of gene targeting in human somatic cells, which led us to believe that rAAV, as a single-stranded virus, may target the human genome in a mechanism similar to plasmid-based targeting vectors, *i.e.*, via two-end, ends-out HR {[5], [6], [11]; Figure S3}.

### The rAAV homology arms remain mostly Intact during random integration

While gene targeting is perforce mediated by homology-directed repair, random integration is believed to be mediated by non-homologous end joining pathways. To test whether gene targeting and random integration produce different molecular products, 38 random clones were recovered and analyzed. 37 of these clones could be amplified by both sets of random integration primers (Figure 1C), indicating that the entire homology arms are almost always retained during random integration. To rule out potential discontinuous homology arm incorporation, a SNP retention analysis was also performed upon the random integration clones. Strikingly, all the SNPs were 100% retained on both arms of the random clones (Figure 1H and Table S3), which confirmed that the homology arms were incorporated intact during random integration. This result is consistent with observations that AAV and rAAV viral∶chromosomal DNA junctions reside almost exclusively within the viral inverted terminal repeats instead of the homology arms during random integration [28]–[30]. The retention of intact viral homology arms during random integration, in contrast to the gradient SNP retention that occurred during gene targeting, unequivocally demonstrated that rAAV gene targeting and random integration are mediated by non-overlapping DSBR pathways.

### rAAV gene targeting occurs predominantly via HR instead of single strand assimilation

**Figure 2:**
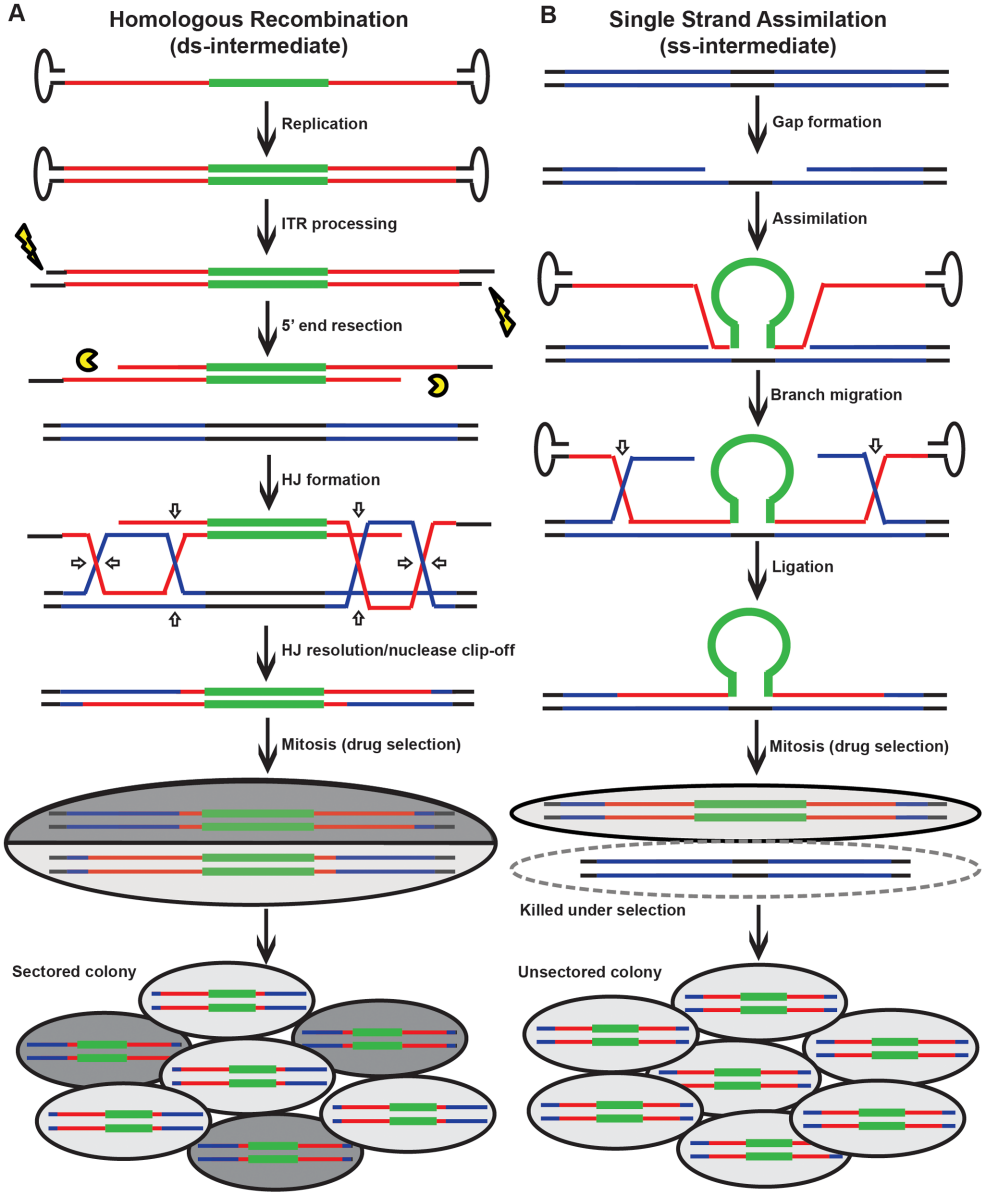
Models for rAAV gene targeting. (A) The HR model. Black, red and blue lines correspond to genomic DNA, viral and genomic homology arms, respectively; the bold green line corresponds to the selection cassette. The vertical arrows imply that the viral DNA becomes double-stranded and the inverted terminal repeats are processed before integrating into the genome. Open arrows represent the sites of HJ cleavage and ligation. A sectored colony is formed during mitosis. (B) The single strand assimilation model. All symbols are as in (A). The virus that anneals to the genomic DNA is single-stranded. In the ensuing mitosis, an unsectored colony is formed under drug selection.

While only single-stranded genomes can be packaged into virions, rAAV becomes double-stranded during replication in the host cell [31]. To determine whether viral ssDNA or dsDNA was the major substrate for gene targeting, a sectoring assay [6], [7], [11] was performed in mismatch repair-deficient HCT116 and DLD-1 cells (Figure 2A and B). If double-stranded viral substrates are used for gene targeting via HR (Figure 2A), both viral strands will be incorporated into a heteroduplex DNA intermediate with unequal length. When this heteroduplex DNA intermediate is resolved by mitosis *in situ*, the two daughter cells will give rise to a heterogeneous colony containing genetically distinct cells that are reciprocally sectored for some of the SNPs on the homology arms (Figure 2A). On the other hand, if gene targeting occurs via single strand assimilation (Figure 2B), a single-stranded viral DNA will be annealed into the heteroduplex DNA. Subsequently, the daughter cell lacking the selection marker will be killed during drug selection, whereas the other will grow into a homogenous colony with all the SNPs unsectored (Figure 2B). Consequently, the relative contribution of HR and single strand assimilation can be expressed as the ratio of the sectored to unsectored colonies produced by rAAV gene targeting.

**Figure 3:**
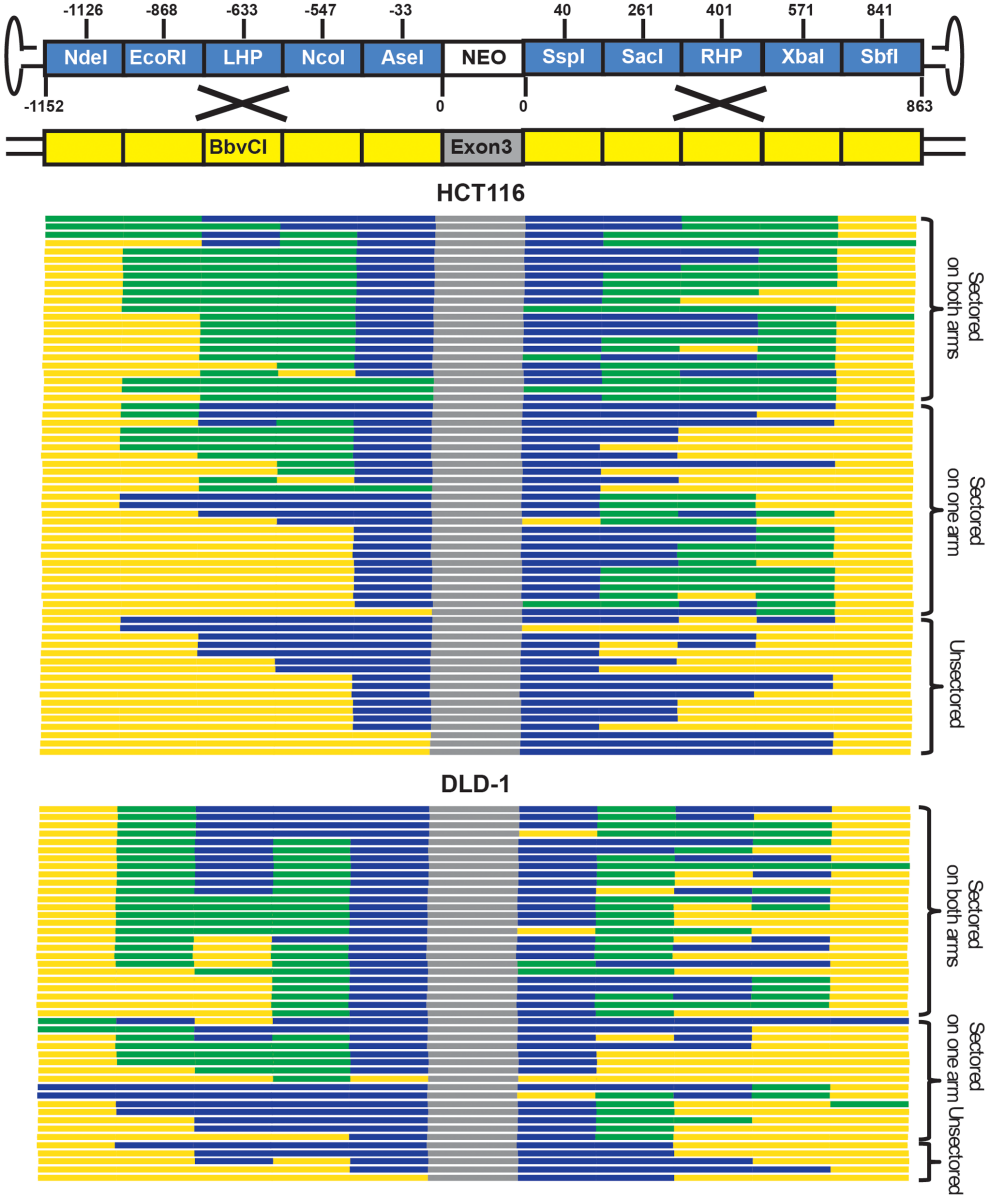
rAAV gene targeting is associated with the formation of sectored colonies. Solid boxes on the top (not to scale) represents diagnostic markers on the virus (blue) and genomic DNA (yellow). The numbers indicate the actual positions of the markers. The NEO cassette and exon 3 of HPRT are indicated in white and grey, respectively. Each line on the bottom corresponds to an independent gene targetin event. The blue, yellow and green segments are color-coded to represent viral, genomic and sectored tracts, respectively. The top and bottom panels show results obtained from HCT116 and DLD-1 cells, respectively.

HCT116 and DLD-1 cells were infected and then allowed to grow into colonies *in situ* in G418- and 6-thioguanine-containing medium. An amount of virus was used to make sure that on average only a single colony was formed in each plate. SNP analysis revealed that 74% and 89% of targeted clones in HCT116 and DLD-1, respectively, were sectored on at least one side of the homology arms (Figure 3 and Tables S4 and S6), consistent with the HR model. Considering that this assay is unable to detect short heteroduplex DNA tracts formed between two neighboring SNPs, this result is likely an underestimation of the actual number of sectored colonies. To rule out the possibility that the sectoring was generated from doublet colonies or two independent single strand assimilation events, 11 clones that were sectored on both arms were subjected to single-cell subcloning. Sequencing analyses demonstrated that 89.6% of the subclones segregated the SNPs with a perfect *trans* configuration (Table S5). Since colonies produced by two independent gene targeting events will have an equal chance to be *trans* or *cis*, the empirically-observed biased *trans∶cis* ratio indicated that most colonies were generated by a single HR event. Thus, in contrast to popular belief, rAAV gene targeting is predominantly mediated by HR in human cells. Nevertheless, since a fraction (26% for HCT116 and 11% for DLD-1) of the targeted clones remained unsectored, we cannot rule out the possible involvement of single strand assimilation as a minor pathway.

### rAAV gene targeting efficiency correlates with the activity of HR

**Figure 4:**
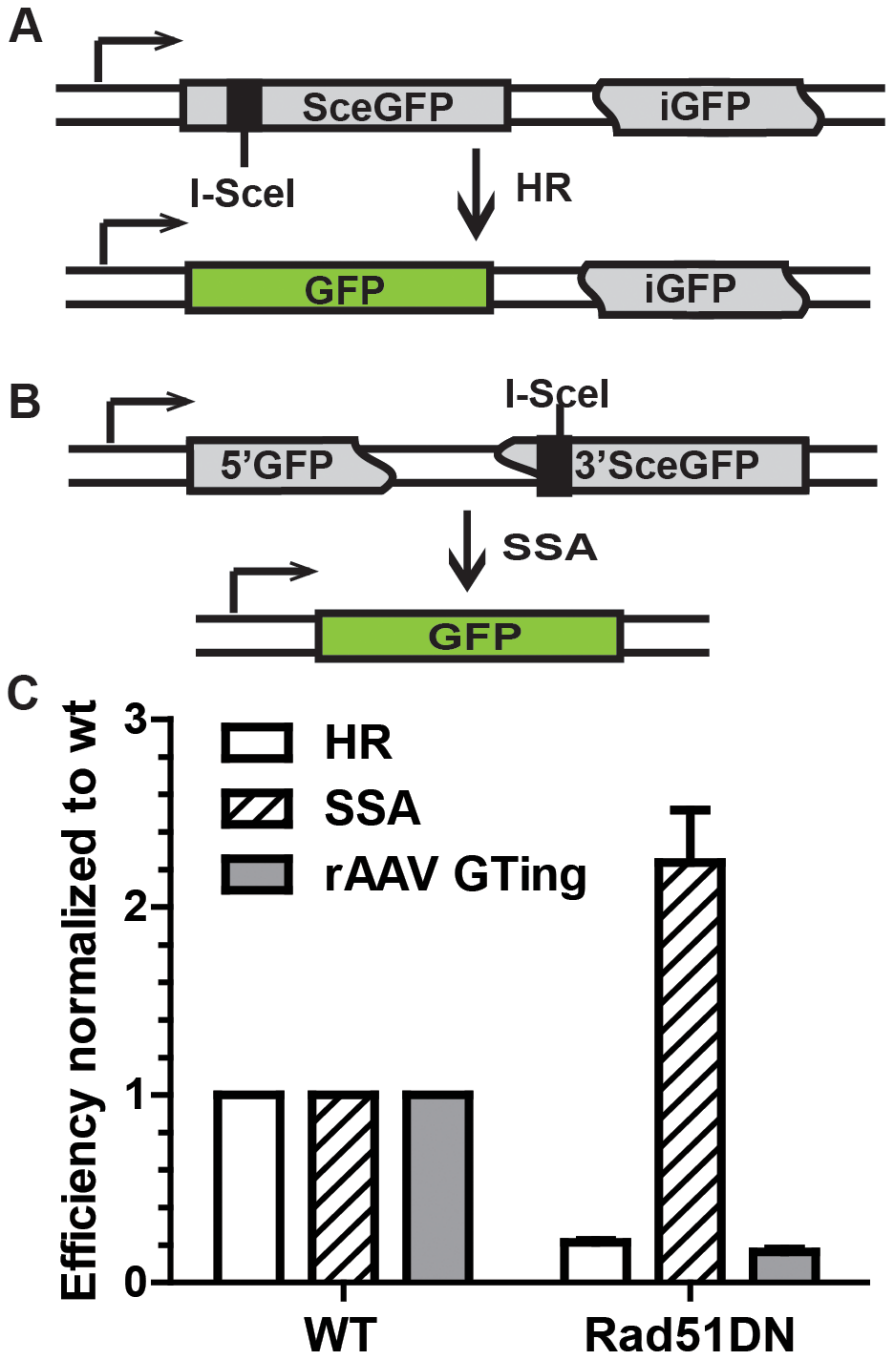
rAAV gene targeting efficiency correlates with HR, and not single strand annealing, activity. (A) The HR assay. SceGFP is a full-length GFP gene disrupted by an I-SceI site. HR between SceGFP and the internal GFP (iGFP) fragment on the same plasmid upon I-SceI digestion restores GFP activity. (B) The single strand annealing assay. 5′GFP and 3′SceGFP are GFP fragments bearing 266 bp of homology. Single strand annealing repair of the I-SceI-induced DSB generates a functional GFP gene. (C) The efficiency of HR, single strand annealing and rAAV gene targeting. The indicated cell lines were analyzed using the HR and single strand annealing assays as well as for rAAV gene targeting. The mean ± SEM of three independent experiments is shown.

To confirm that rAAV gene targeting efficiency correlated with HR, and not single strand assimilation, activity, we transfected HCT116 cells with Rad51K133A, a dominant negative form of Rad51 reported to reduce HR and concomitantly elevate single strand annealing [23]. Using episomal reporters for either HR (Figure 4A) or single strand annealing (Figure 4B), we confirmed that expression of the dominant negative indeed reduced HR and increased single strand annealing in HCT116 cells (Figure 4C). Importantly, the rAAV targeting efficiency at the HPRT locus was reduced by 6.2-fold upon Rad51K133A transfection, which correlated well with the reduced HR activity and not the increased single strand annealing activity in these cells (Figure 4C). Thus, consistent with the sectoring assay, this result further confirmed that rAAV gene targeting is mediated predominantly by HR instead of single strand assimilation in human cells.

### rAAV gene targeting inverts to an ends-in mechanism in the presence of DSBs

**Figure 5:**
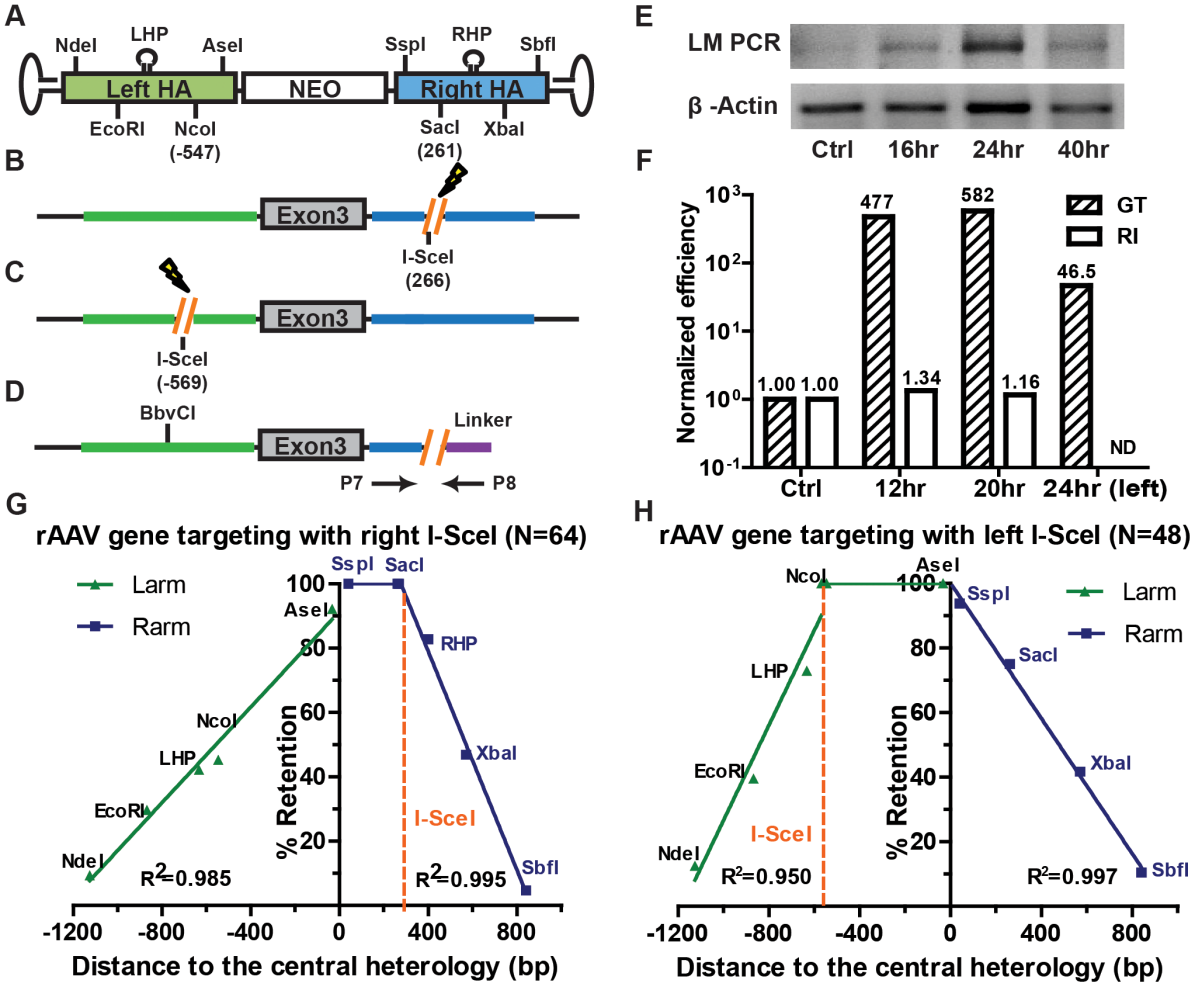
Chromosomal DSBs shift the SNP retention signature of rAAV gene targeting. (A) The rAAV targeting vector. See the legend to Figure 1A for details. (B) The HPRT locus. An I-SceI restriction site (orange) was knocked-in to the chromosome at the indicated corresponding vector position. The lightening bolt denotes that DSBs can be induced upon I-SceI expression. (C) The HPRT locus; left side analysis. All symbols as in (B). (D) Scheme for the LM PCR. A linker with a 5′ I-SceI overhang was ligated to the 3′ end of the genomic I-SceI-generated break. The presence of a ligation product was quantitated with the primers indicated by arrows. (E) I-SceI-induced chromosomal DSBs can be detected within 24 hr of I-SceI expression. A gel electrophoresis analysis of the PCR products generated using genomic DNA isolated at the indicated times following I-SceI expression. β-Actin was used as a loading control. (F) The efficiency of I-SceI-induced rAAV gene targeting. Cells were infected with rAAV without (Ctrl) or 12 or 20 hr (for the right side) or 24 hr (for the left side) after I-SceI expression. The gene targeting and random integration frequencies were normalized to the no I-SceI control. (G) The SNP retention signature of I-SceI-induced rAAV gene targeting; right side. The dotted orange line indicates the position of the I-SceI site. All other symbols are defined in Figure 1A. (H) The SNP retention signature of I-SceI-induced rAAV gene targeting; left side. All symbols are as in (G).

Spontaneous endogenous DSBs occur around 10 times per mammalian cell per day [8]. The likelihood that one of these DSBs must be introduced near a target locus in order for rAAV-mediated gene targeting to occur is statistically improbable. rAAV gene targeting must, therefore, employ a mechanism that is independent of the formation of chromosomal DSBs (Figure 2A). Nevertheless, rAAV gene targeting can be stimulated dramatically by the presence of chromosomal DSBs near the target locus [32]–[34]. The mechanistic basis for this increase is, however, not understood. To investigate this issue, rAAV was used to “knock-in” an *I-SceI* enzyme recognition sequence onto the X chromosome at a site that corresponded to a position (nt 266), just to the right of the *SacI* (nt 261) site, on the right homology arm of the HPRT rAAV targeting vector (Figure 5A and B and Figure S4). After transfection with an *I-SceI* expression plasmid, chromosomal DSBs were quantified by ligation-mediated PCR {[35]; Figure 5D}. DSBs were detectable 16 hr after transfection, and peaked ∼24 hr after transfection (Figure 5E). Accordingly, rAAV infections were performed either 12 or 20 hr after *I-SceI* transfection in an attempt to coordinate the viral infection with the chromosomal DSB induction. The absolute gene targeting efficiency increased by 477- and 582-fold, respectively, in the presence of *I-SceI* (Figure 5F), which was consistent with previous reports [32]–[34]. The random integration frequency was virtually unperturbed by the expression of *I-SceI* (Figure 5F). The retention of viral SNPs was then analyzed in 64 targeted clones. Strikingly, the *SspI* and *SacI* sites on the right homology arm were both retained at 100% frequency (Figure 5G and Table S7), which was in stark contrast to the linear gradient of SNP loss in non-DSB-induced gene targeting (compare Figure 5G with Figure 1E and F). The SNPs to the right of the *I-SceI* site (the RHP, *XbaI* and *SbfI*) were lost in a sharper, but nonetheless linear, gradient (Figure 5G). To confirm this finding, we constructed another cell line in which rAAV was used to knock-in an *I-SceI* enzyme recognition sequence into the X chromosome at a site that corresponded to a position (nt −569), just to the left of the *NcoI* (nt −547) site, on the left homology arm of the HPRT rAAV targeting vector (Figure 5A and C and Figure S4). The rAAV gene targeting frequency was also elevated by concomitant *I-SceI* expression (Figure 5F). The retention of viral SNPs was then analyzed in 48 targeted clones. In a strikingly mirrored fashion, the *AseI* and *NcoI* sites on the left homology arm were both retained at 100% frequency, while the SNPs to the left of this region (the LHP, *EcoRI*, *NdeI*) were lost in a linear gradient (Figure 5H and Table S8).

**Figure 6:**
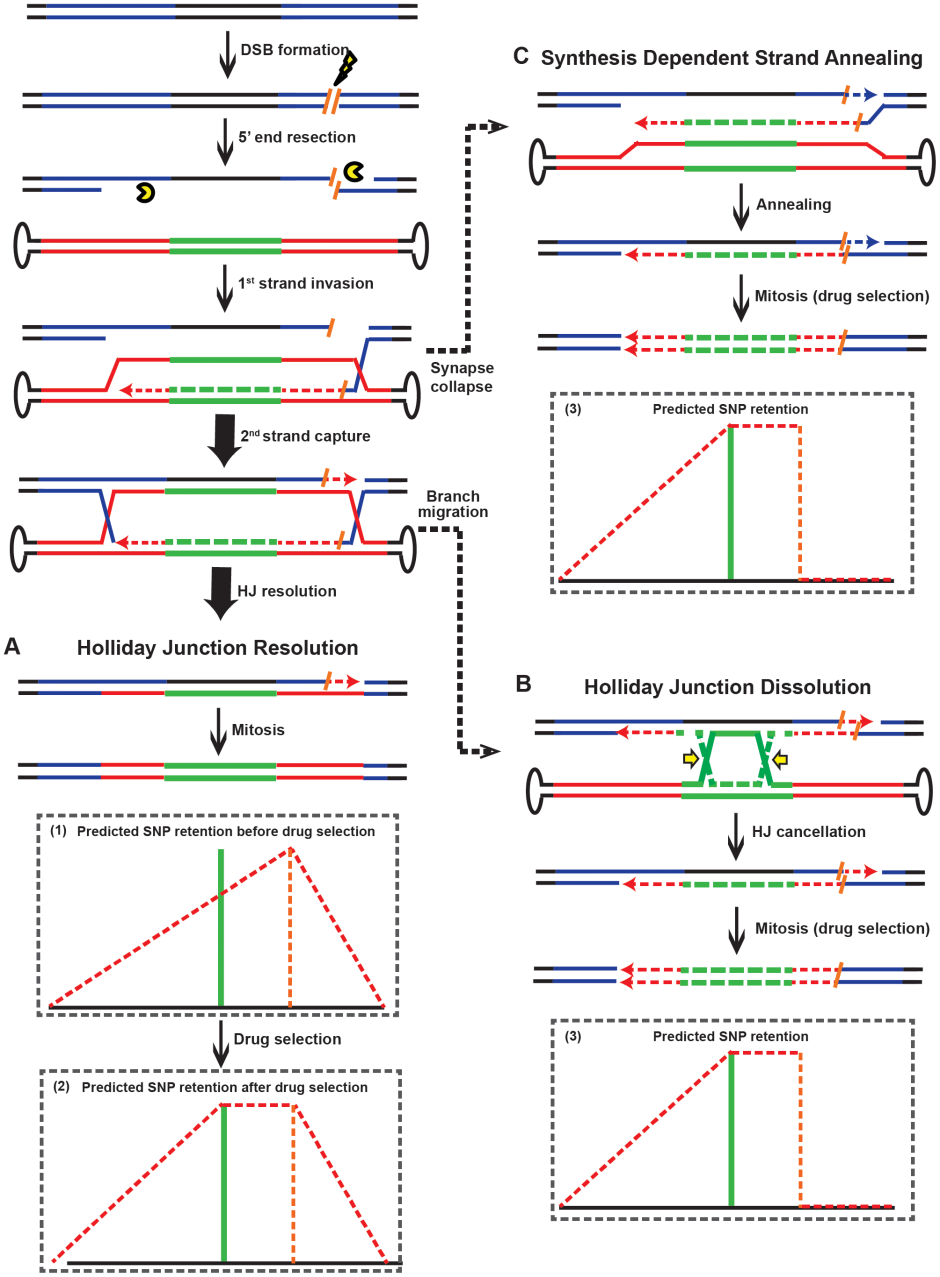
Models for rAAV gene targeting in the presence of DSBs. (A) rAAV gene targeting in the presence of DSBs. Dotted lines and arrowheads correspond to de novo DNA synthesis, which is color-coded to match the templates. Orange slashes represent half I-SceI sites and the lightening bolt represents I-SceI-induced cleavage. All other symbols are as in Figure 2. The chromosomal ends are processed and invade the viral DNA in an ends-in configuration. Two predicted SNP retention patterns (minus and plus drug selection, respectively) are cartooned as (1) and (2), respectively. (B) Holliday Junction dissolution. Branch migration forces the HJs towards the drug selection cassette and the HJ is cancelled. The predicted SNP retention pattern is cartooned in (3). (C) Synthesis dependent strand annealing. If the synapsed structure shown in (A) collapses, recombination can still occur by SDSA. This mechanism, like HJ dissolution (B), predicts the SNP retention pattern shown in (3).

The plateaued SNP retention curves observed in these 2 experiments are predicted from an “ends-in” gene targeting model in which recombination is initiated not by the vector DNA but by the broken chromosome (Figure 6A). In contrast to non-DSB-mediated rAAV gene targeting where the viral DNA “attacks” the unbroken chromosome in an ends-out configuration (Figure 2A), in DSB-induced gene targeting the broken chromosomal ends are instead processed and invade the virus in an ends-in configuration (Figure 6A and Figure S3). Without drug selection, the random distribution of HJ resolution would produce a gradient retention curve peaking at the *I-SceI* site (Figure 6A-1; cartooned for the rightward *I-SceI* site). However, because G418 selection was imposed, any HJs that were resolved between the *I-SceI* site and the selection cassette would have been lost. Consequently, the initiation of recombination with the chromosomal *I-SceI*-restricted ends and the requirement for the retention of the viral selection cassette precisely explain the SNP retention pattern that we obtained (compare Figure 5G with Figure 6A-2). In summary, the introduction of a chromosomal DSB inverts the process of gene targeting such that the viral DNA becomes the “attackee” instead of the attacker.

These data also established an important corollary. Three pathways can act independently to resolve an HR intermediate: HJ resolution (the DSBR model), HJ dissolution and synthesis-dependent strand annealing (SDSA) {[36], [37]; Figure 6}. HJ resolution features the formation and resolution of double HJs (Figure 6A) whereas inward branch migration of the HJs can cause HJ dissolution (Figure 6B). Alternatively, in SDSA the synapse collapses before the formation of the second HJ (Figure 6C). SDSA is believed to be the major pathway of mitotic recombination in yeast and plants [38], [39]. It is also the preferred pathway of repairing an *I-SceI*-induced DSB in mouse and human cells [40]. Importantly, both the SDSA and HJ dissolution models predict the retention of one half of the *I-SceI* site and the loss of all of the SNPs rightward of the right *I-SceI* site (Figure 6-3), or leftward of the left *I-SceI* site (not shown), a minor pattern that was observed in only 17% of the clones (Table S7). Collectively, these results suggest that although SDSA may be the major pathway for recombination in mitotic cells, HJ resolution (the DSBR model) is the predominant form of HR that leads to gene targeting in human somatic cells.

### The anti-recombination activity of mismatch repair strongly inhibits gene targeting

**Figure 7:**
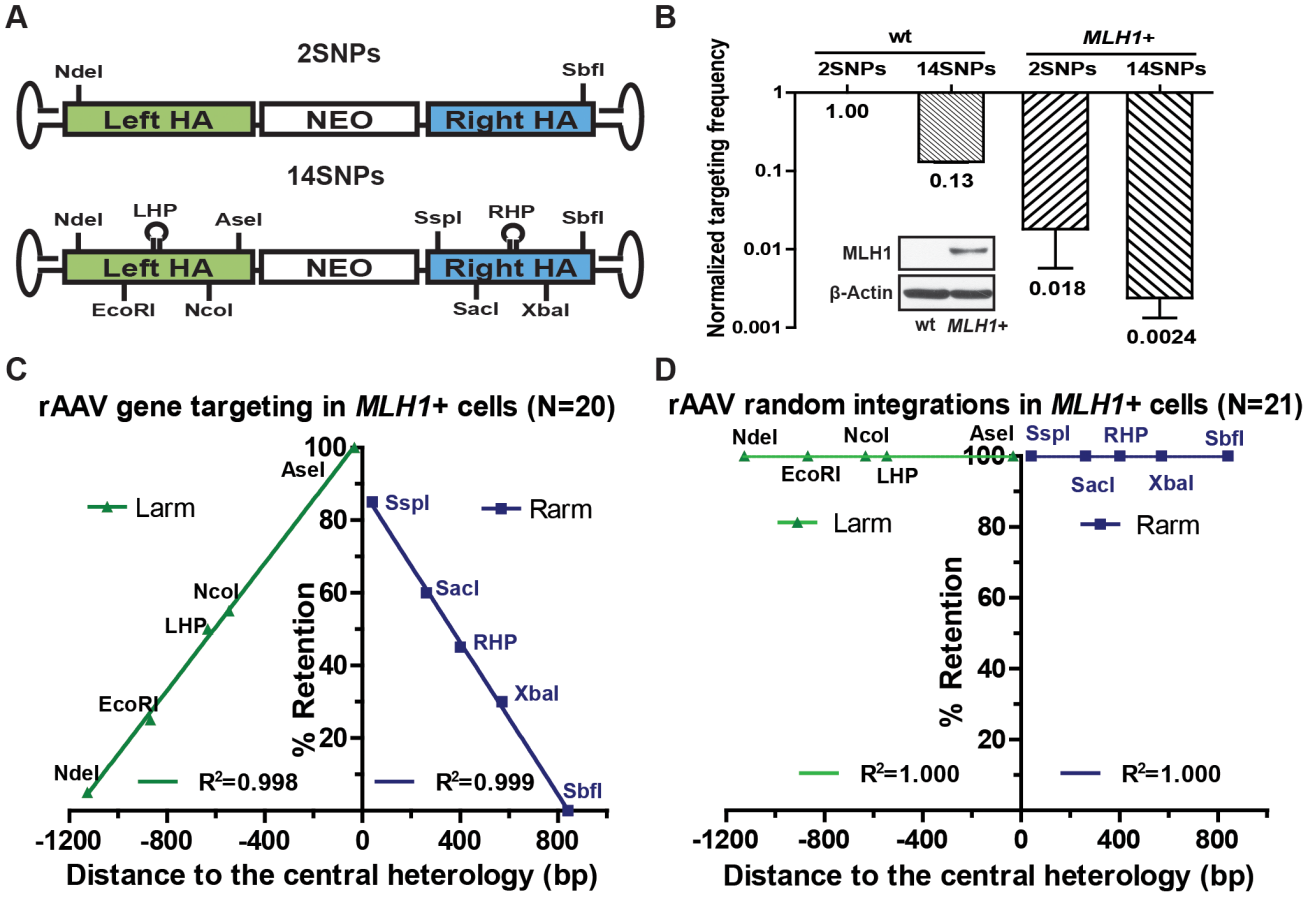
rAAV gene targeting is suppressed in a mismatch repair-proficient background. (A) The rAAV targeting vectors. All symbols as in Figure 1A. 2SNPs and 15SNPs indicate the total number of mismatches in the vectors. (B) Effects of mismatches and the host mismatch repair status on rAAV gene targeting. The rAAV gene targeting efficiency is expressed as the ratio of correctly targeted clones divided by the sum of the correctly targeted plus the randomly integrated clones. All results are normalized to the parental (MLH1^−^) cell line. The mean ± SEM of three independent experiments is shown. The MLH1 expression in the parental and MLH1^+^ cell lines is shown in the inserted Western blot panel. b-actin was used as a loading control. (C and D) The SNP retention signature of rAAV gene targeting and random integration respectively in the mismatch repair (MLH1^+^) -proficient background. All symbols are as in Figure 1E.

Since the SNPs engineered into the rAAV targeting vector generated mismatches in the heteroduplex DNA intermediate, we wished to assess if they were sensitive to mismatch repair. Thus, another rAAV targeting vector was constructed with only 2 SNPs and tested in the parental HCT116 (mismatch repair-deficient) cell line (Figure 7A). The targeting efficiency was 7.5-fold higher compared to the original vector, which contained 15 SNPs (Figure 7B). These data indicated that the presence of mismatches deleteriously affected gene targeting even in a mismatch repair-reduced background, a result that can be attributed to the residual mismatch repair activity present in this cell line [41]. To further address the role of the mismatch repair system, gene targeting was performed in an mismatch repair-proficient variant (MLH1^+^), in which the mutated MLH1 gene in HCT116 cells was corrected by rAAV-mediated knock-in (Figure 7B, inset). Targeting efficiency decreased by more than 50-fold in MLH1^+^ cells for each of the vectors respectively compared with the isogenic MLH1-defective parental line (Figure 7B). Collectively, these data demonstrated that the mismatch repair gene MLH1 exerts a strong inhibitory effect on gene targeting [7], [42].

Mismatch repair has two well-documented activities. One is as a “spell-checker” to correct post-replication mismatches in DNA and the other is as an “anti-recombinase”, by impeding the formation of homeologous heteroduplex DNA [42], [43]. To assess which of these two activities was responsible for reducing gene targeting, 20 targeted clones were recovered — despite the extremely low targeting efficiency in MLH1^+^ cells — and analyzed for SNP retention (Figure 7C and Table S9). Importantly, the SNP retention curve for MLH1^+^ cells was indistinguishable from the parental (MLH1^−^) linear retention curve (compare Figure 1E and F with Figure 7C). Moreover, the hairpins, which are refractory to the spell-checking activity of mismatch repair [12], [41], were retained at the same frequency as is predicted by the linear regression of other SNPs, which are substrates for spell-checking. Finally, the percentage of discontinuous gene conversion tracts (a hallmark of spell-checking) did not change significantly in the mismatch repair-proficient, compared to the mismatch repair-deficient, background (compare Table S9 with Table S1, respectively). These results demonstrated that the presence of MLH1 exercised no detectable spell-checker activity upon the mismatches in the heteroduplex DNA intermediate and implied that the large, negative impact of MLH1 on gene targeting was instead due to anti-recombination activity of mismatch repair [7], [42], [43]. Finally, to test whether the mismatch repair system affects random integration, 22 G418-resistant 6-thioguanine-sensitive clones were recovered from the MLH1^+^ background and analyzed for SNP retention. All but one of them could be amplified using the random integration primers, and once again, 100% of the viral SNPs were retained (Figure 7D and Table S10), which is consistent with the observation that mismatch repair does not affect non-homologous end joining [43].

## Discussion

### rAAV uses the DSBR pathway of HR for gene targeting

Although rAAV is widely used in laboratory and clinical studies, the mechanism of rAAV-mediated gene targeting has remained obscure. Since rAAV is packaged exclusively as a single-stranded virus, several reports have suggested that rAAV gene targeting is mediated by single strand assimilation [17], [18]. Moreover, the single strand assimilation model is supported by indirect evidence that minute virus of mouse, a related parvovirus, shows a strand-specific bias in gene targeting [44]. Our data, however, using three lines of evidence demonstrate that rAAV gene targeting is mediated by the DSBR model of HR using double-stranded viral substrates: (1) rAAV gene targeting produces the same SNP retention curve as that of plasmid-based gene targeting, which is dictated by two-end, ends-out HR [6], [11]. (2) rAAV gene targeting is associated with the formation of sectored colonies in a *trans* configuration, which is characteristic of the DSBR model. (3) rAAV gene targeting frequency correlated with HR, and not single strand annealing, activity through the use of Rad51K133A transfections. These results demonstrate that rAAV has to become double-stranded — either by host DNA polymerases or by annealing of the plus and minus viral strands — before targeted integration can occur.

What is less clear, given that rAAV uses the same mechanism as linearized plasmid-based targeting vectors, is why rAAV targets human cells so much more robustly. We suggest that there are several viral elements of rAAV that may positively influence gene targeting. For example, the capsid proteins may facilitate virus transduction and nuclear trafficking via interaction with cellular receptors [45] to generate higher nuclear concentrations of the viral DNA versus transfected DNA. In addition, the hairpin-structured inverted terminal repeats may serve as physical barriers to protect the ends of the viral genome from nuclease degradation during nuclear trafficking. An alternative possibility that we favor is that the inverted terminal repeats may facilitate the formation of active recombination substrates. Thus, besides the recombinogenic linear viral dsDNA, infected cells also contain a mixture of viral ssDNA along with circular and concatemerized dsDNA [46]. Our ends-out recombination model requires that both ends of the viral genome are accessible to exonuclease resection, which means that the linear, monomeric double-stranded viral genomes are the only active substrates that can be used for gene targeting. Since the inverted terminal repeats suppress the intra- and intermolecular recombination that generates viral circular and concatemerized dsDNA [47], they may facilitate gene targeting by favoring the existence of the active recombination substrates. On the contrary, plasmid-based gene targeting vectors may be efficiently inactivated by circularization or concatemerization before gene targeting can occur. Needless, to say, none of these hypotheses are mutually exclusive and they may act synergistically to enhance rAAV gene targeting.

### The rAAV gene targeting system as a model to study HR in human somatic cells

The locations of crossovers are determined by the initial positions of HJ formation and branch migration activity. Comprehensive gene conversion tract analyses have been performed in yeast, flies and mouse embryonic stem cells, which revealed an exponential retention of donor sequence during meiotic and mitotic HR {[7], [20]–[22]; Figure S2}. These studies indicated that the crossovers were more likely to occur near the initiation site of strand invasion, probably as a result of branch migration. Although similar studies have been undertaken in mammalian systems [10]–[13], [40], [48] the generality of the conclusions were restricted by the limited scale of the data. Taking advantage of the high targeting efficiency of rAAV, we performed a SNP retention analysis for non-DSB (Figure 1E and F) and DSB-induced (Figure 5G and H) gene targeting in human cells with unprecedented resolution. In contrast to previous studies, we obtained a sharp linear retention curve, indicating that crossovers are evenly, and not exponentially, distributed along the homology arms. We further confirmed the generality of the SNP retention curve using plasmid-based gene targeting, although on a smaller scale (Figure 1G). Assuming that each segment of the homology arms has the same tendency to initiate strand exchange [49], we propose that the linear SNP retention curve in human cells is shaped primarily by the even distribution of HJ formation and is minimally impacted by branch migration. It should be noted that alternative scenarios are possible. For example, rather than formation of a second HJ (Figure 2A), the distal ends could be resolved by cleavage with structure-specific endonucleases such as XPF/ERCC1 [50], [51]. Our linear SNP retention curve favors the former scenario, but we cannot rule out the latter possibility.

Branch migration reshapes the distribution of crossovers and determines the amount of genetic information exchanged during HR. Interestingly, bacterial RecA and its mammalian Rad51 homologs facilitate branch migration in different directions: RecA moves the HJs away from DSBs to encourage the exchange of genetic material in bacteria, whereas in lower eukaryotes, Rad51 shifts the HJs towards DSBs to minimize gene conversion tracts [52]. Our results are consistent with the *in vitro* observation that the branch migration activity of human Rad51 is substantially lower than its yeast counterpart [52], which suggests that human cells may have adopted an energy-saving strategy to repair somatic DSBs by HR without suppressing the amount of genetic material in the exchange.

An additional insight from our studies is the demonstration that meganucleases stimulate gene targeting by promoting chromosome-initiated, ends-in recombination. Creating a DSB in a target locus increases the frequency of gene targeting by 2 to 3 orders of magnitude {[32]–[34], [53]; Figure 5F}, which makes artificial meganucleases promising tools for genetic engineering. The mechanism for the enhanced gene targeting frequency was, however, unknown. Importantly, our chromosome-initiated ends-in recombination model immediately provides an explanation for this profound enhancement. As discussed earlier, the viral DNA inside an infected cell can exist as linear, circular or concatemeric species and only the former of these is proficient for ends-out recombination. Since the majority of the viral genomes are converted into circular or concatemeric forms by cellular DSBR pathways shortly after infection [31], [46] the efficiency for spontaneous gene targeting is low. In contrast, in DSB-induced gene targeting, the broken chromosome ends can invade all of these exogenous species to initiate HR. Also, this ends-in recombination involves the resolution of only two — instead of the four — HJs required for the ends-out model. These differences may together contribute to the orders of magnitude increase in targeting efficiency.

The demonstration of two modes for gene targeting explains an additional conundrum in the field. Thus, by themselves, single-stranded oligonucleotides are poor donors for gene targeting in mammalian cells [54]. Paradoxically, with the development of artificial meganucleases, zinc finger nucleases {ZFNs; [55]}, transcription activator like effector nucleases {TALENs; [56]} and clustered regularly interspaced short palindromic repeats∶CRISPR-associated {CRISPR-Cas; [57]} reagents to mediate gene targeting, there has been a spate of recent papers demonstrating that single-stranded oligonucleotides can be efficiently used to facilitate HR in the presence of a DSB {*e.g.*, [58], [59]}. This “paradox” however, is precisely what our data would predict: by itself, an single-stranded oligonucleotide would need to engage one of the minor HR pathways (*e.g.*, single strand annealing) to initiate gene targeting. In contrast, following a meganuclease-induced DSB, the resulting chromosomal ends should efficiently and productively be able to interact with an accompanying single-stranded oligonucleotide.

Finally, our data demonstrate that the DSBR model is the preferred pathway of HR leading to gene targeting in human cells. The DSBR model has become the paradigm of HR [19], which is characterized by the formation of double HJs and resolution by resolvases (Figure 6A). However, this model has been challenged by the fact that mitotic recombination is infrequently associated with crossovers. SDSA emerged as an alternative model [60], in which the invading strand anneals back with its original partner after *de novo* DNA synthesis without the formation of HJs (Figure 6C). In yeast, plants and mammals, a large body of evidence suggests that SDSA is the preferred pathway of mitotic recombination [38]–[40]. There is less convincing data that SDSA is utilized for gene targeting, although it should be noted that the ERCC1/XPF nuclease complex, which has documented roles in single strand annealing and in SDSA [51], can impact the process of mammalian gene targeting as well [61]. Our SNP retention analysis in the presence of a chromosomal DSB, however, indicated that the bulk of the gene targeting products are generated by the DSBR model, at least when the conversion of a large drug selection marker is required (Figure 6A). This result strongly argues that — in human somatic cells — gene targeting is most accurately described by the DSBR model.

*In toto*, it should also be emphasized that there are a multitude of differences, some subtle and some not so that distinguish human gene targeting from that described in other systems: *e.g.*, 1) the even distribution of crossovers in the homology arms, 2) the preferred use of DSBR versus SDSA or HJ dissolution, and 3) the preferential use of broken chromosomal ends over the ends of exogenous DNA. Understanding the mechanistic underpinnings of these differences will be critical to improve the efficacy of gene targeting for therapeutic purposes.

## Materials and Methods

### Cell culture

The HCT116 and DLD-1 cell lines were cultured in McCoy's 5A medium supplemented with FBS, L-glutamine, penicillin and streptomycin with 5% CO_2_ at 37°C.

### Cell lines and plasmids

The HCT116 cell line was obtained from ATCC. The MLH1^+^ cell line was provided by Horizon Discovery, Ltd. The DLD-1 cell line was obtained from Dr. D. Largaespada. The DR-GFP and SA-GFP reporter plasmids were obtained from Dr. M. Jasin and the Rad51K133A expression vector was obtained from Dr. J. Stark [23].

### Viruses

Briefly, the left and right homology arms were amplified by PCR from HCT116 genomic DNA. Viral SNPs were introduced using a QuickChange site-directed mutagenesis kit. The arms were then joined with a drug selection cassette using fusion PCR and the resulting product was ligated to a pAAV backbone. All virus packaging and infections were performed as described [15].

### Vector-borne marker analysis

Genomic DNA was Isolated and the homology arms of the GT and RI clones were amplified by diagnostic PCRs (Figure 1C). The retention of the vector-borne markers was analyzed first by restriction enzyme digestion and then confirmed by sequencing.

### Repair assays

Briefly, cells were subcultured in 6-well tissue culture plates. The next day, the cells were transfected with 0.5 µg mCherry, 1.0 µg of an *I-SceI* expression plasmid and 1.0 µg DR-GFP or SA-GFP assay substrates. GFP and mCherry expression was then analyzed 48 hr post transfection using flow cytometry. The repair efficiency was calculated as the percentage of GFP and mCherry doubly positive cells divided by the mCherry-positive cells. For the Rad51DN experiment, an additional 1.0 µg of the Rad51K133A expression plasmid was transfected as well.

### Targeting efficiency assay

Briefly, cells were subcultured in 6-well tissue culture plates on day 1. On day 2, 100 µl of the appropriate viral stock was added to the wells. On day 4, the cells were counted and aliquoted into 10 cm tissue culture dishes for drug selection. The plates were supplemented with 1 mg/ml G418 or 0.5 mg/ml G418 plus 5 µg/ml 6-thioguanine for 12 days. The gene targeting and random integration efficiencies were calculated as the number of G418-resistant 6-thioguanine-resistant and G418-resistant 6-thioguanine-sensitive colonies per 10^6^ cells, respectively. Results were averaged from 7 plates. For the Rad51DN experiment, cells were transfected with 2.5 µg Rad51K133A expression plasmid 48 hr before infection. For the *I-SceI* experiments, cells were transfected with 2.5 µg of an *I-SceI* expression plasmid 12 or 20 hr before infection.

### Ligation-mediated PCR

Genomic DNA was isolated at the designated times after *I-SceI* induction. DNA (1 µg) was ligated with 100 pmol of adaptors at 16°C overnight. PCR was performed at the linear stage using a 25 ng ligation product with the primers illustrated in Figure 5D. β-actin primers were used as loading control.

## Supporting Information

Figure S1 Overview of rAAV production and gene targeting. A cartoon strategy for rAAV virus production and gene targeting is shown. At the top left are cartooned three plasmids that contain *i*) the AAV viral genes: Rep (orange rectangle) for replication and Cap (purple rectangle) for capsid, *ii*) the rAAV vector containing a backbone (purple lines), the ITRs (bubbles), HAs (red lines) and the selection cassette (green box) and *iii*) the plasmid encoding adenoviral (Ad) helper functions (gray rectangle). These three plasmids are triple transfected into AAV293 cells, where the viral and Ad helper proteins are expressed and facilitate the replication of the viral DNA. Virions (hexagons), containing single-stranded rAAV, are subsequently collected from the supernatant of these cells and used to infect a target cell. In the target cell, the rAAV once again assumes a double-stranded form and facilitates gene targeting at your favorite gene (YFG).
(TIF)

Figure S2 Different SNP retention patterns of lower organisms and human somatic cells. In all organisms, the process of GTing appears to be initiated with the same steps: strand resection (PacMan) and HJ formation. The blue lines represent chromosomal DNA, the red lines viral DNA and the green lines the selection cassette. In yeast, flies and murine ES cells, the process of branch migration (orange arrows) pushes the HJs towards each other (left), whereas in human cells this process is apparently negligible. HJ resolution (small white arrows) of these structures generates either an exponential SNP retention curve (in the presence of inward branch migration, left) or a linear SNP retention curve (in the absence of branch migration, right). The amount of genetic information that is exchanged is correspondingly restricted (left) or enlarged (right).
(TIF)

Figure S3 Definition of ends-out and ends-in recombination. A cartoon of a chromosome (blue oval with yellow circular centromere) and a gene targeting vector (red lines) containing a drug selection cassette (green rectangle) is shown. In the ends-out recombination, the 3′ ends of the targeting vector invade (black lines with arrowheads) — in directions opposite to each other — the chromosome in separate HR reactions. In the spontaneous ends-in recombination, the 3′ ends of the targeting vector invade the chromosome in directions facing each other. In the chromosomal DSB-induced ends-in recombination, the broken chromosomal ends (jagged blue ovals) invade/anneal to the targeting vector in directions facing each other.
(TIF)

Figure S4 Construction of a human cell line containing an *I-SceI* site imbedded in the HPRT locus. A cartoon of a rAAV *I-SceI* knock-in targeting vector is shown on the top line. The *I-SceI* recognition site is shown a double-hatched line. A cartoon of the relevant portion of the HPRT locus (horizontal line with a rectangular exon 3) is shown on the line below. Following correct GTing, both the *I-SceI* recognition site and the NEO drug resistance gene are integrated at the HPRT locus. Following Cre recombination, the NEO gene is removed and a solo LoxP scar (shaded triangle) remains.
(TIF)

Table S1 SNP retention of rAAV gene targeting colonies in parental HCT116 cells.
(PDF)

Table S2 SNP retention of rAAV random integration colonies in parental HCT116 cells.
(PDF)

Table S3 SNP retention of plasmid-based gene targeting in parental HCT116 cells.
(PDF)

Table S4 The sectoring assay in parental HCT116 cells.
(PDF)

Table S5 Subcloning of colonies from Table S4 that are sectored on both arms.
(PDF)

Table S6 The sectoring assay in parental DLD-1 cells.
(PDF)

Table S7 SNP retention of *I-SceI*-induced rAAV gene targeting on the right homology arm.
(PDF)

Table S8 SNP retention of *I-SceI*-induced rAAV gene targeting on the left homology arm.
(PDF)

Table S9 SNP retention of rAAV gene targeting colonies in *MLH1^+^* HCT116 cells.
(PDF)

Table S10 SNP retention of rAAV random integration colonies in *MLH1^+^* HCT116 cells.
(PDF)
