# Supplementary figures and images for: The Mechanism of Gene Targeting in Human Somatic Cells
Source: PLoS Genet. 2014 Apr 3;10(4):e1004251. doi: 10.1371/journal.pgen.1004251 (PMC3974634; doi:10.1371/journal.pgen.1004251)

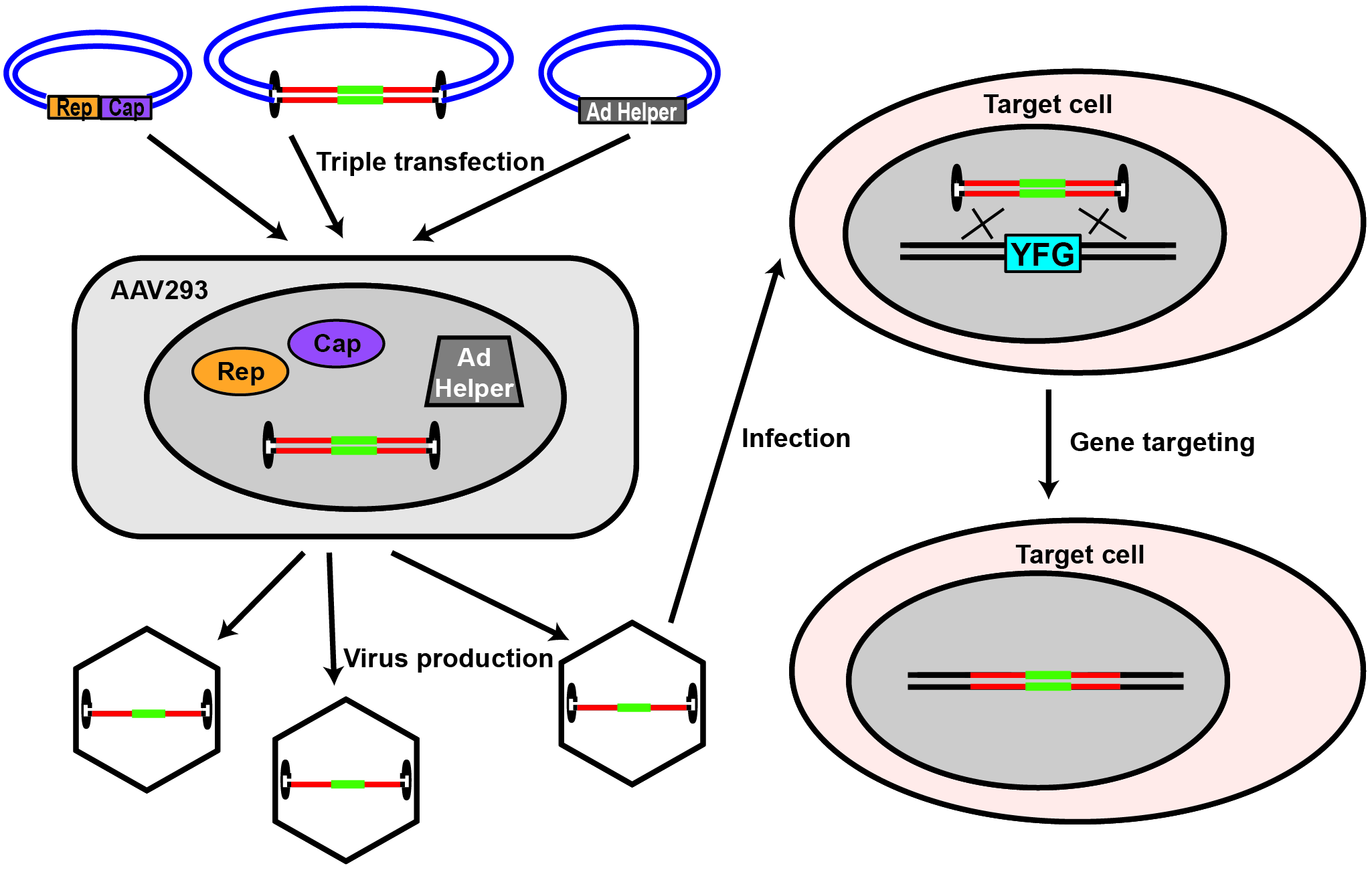

Supplement: Figure S1 — Overview of rAAV production and gene targeting. A cartoon strategy for rAAV virus production and gene targeting is shown. At the top left are cartooned three plasmids that contain i) the AAV viral genes: Rep (orange rectangle) for replication and Cap (purple rectangle) for capsid, ii) the rAAV vector containing a backbone (purple lines), the ITRs (bubbles), HAs (red lines) and the selection cassette (green box) and iii) the plasmid encoding adenoviral (Ad) helper functions (gray rectangle). These three plasmids are triple transfected into AAV293 cells, where the viral and Ad helper proteins are expressed and facilitate the replication of the viral DNA. Virions (hexagons), containing single-stranded rAAV, are subsequently collected from the supernatant of these cells and used to infect a target cell. In the target cell, the rAAV once again assumes a double-stranded form and facilitates gene targeting at your favorite gene (YFG). (TIF) [file pgen.1004251.s001.tif]

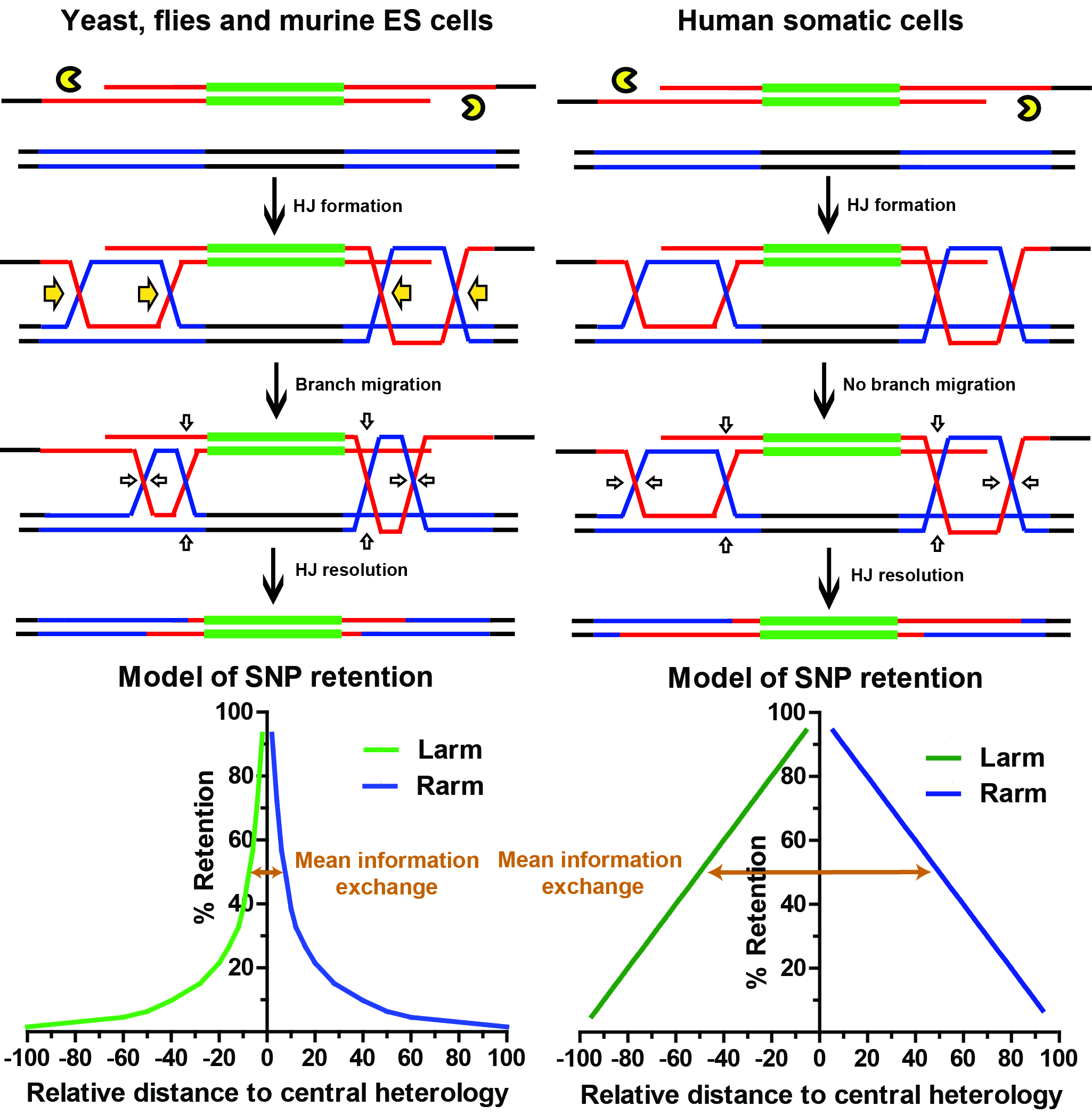

Supplement: Figure S2 — Different SNP retention patterns of lower organisms and human somatic cells. In all organisms, the process of GTing appears to be initiated with the same steps: strand resection (PacMan) and HJ formation. The blue lines represent chromosomal DNA, the red lines viral DNA and the green lines the selection cassette. In yeast, flies and murine ES cells, the process of branch migration (orange arrows) pushes the HJs towards each other (left), whereas in human cells this process is apparently negligible. HJ resolution (small white arrows) of these structures generates either an exponential SNP retention curve (in the presence of inward branch migration, left) or a linear SNP retention curve (in the absence of branch migration, right). The amount of genetic information that is exchanged is correspondingly restricted (left) or enlarged (right). (TIF) [file pgen.1004251.s002.tif]

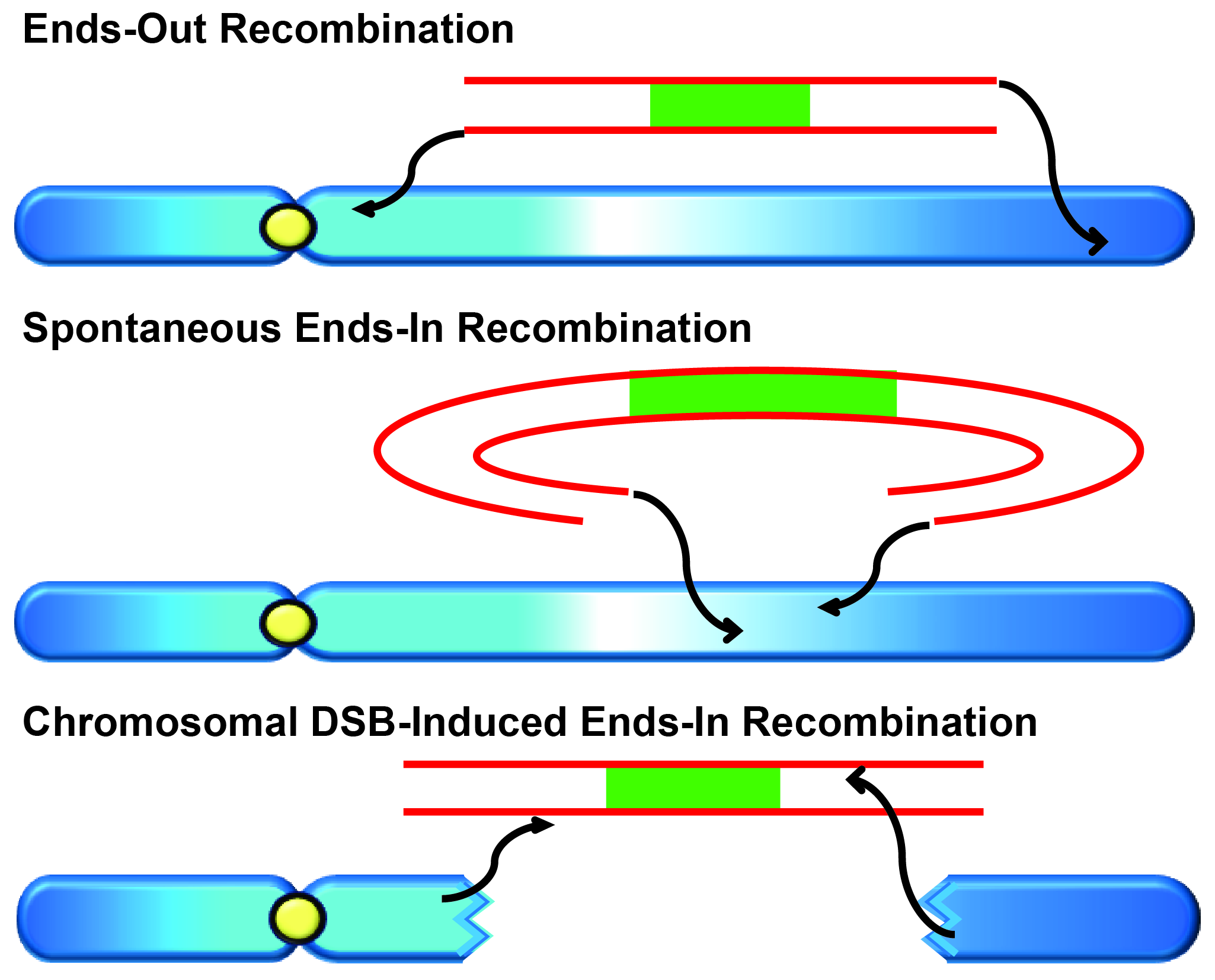

Supplement: Figure S3 — Definition of ends-out and ends-in recombination. A cartoon of a chromosome (blue oval with yellow circular centromere) and a gene targeting vector (red lines) containing a drug selection cassette (green rectangle) is shown. In the ends-out recombination, the 3′ ends of the targeting vector invade (black lines with arrowheads) — in directions opposite to each other — the chromosome in separate HR reactions. In the spontaneous ends-in recombination, the 3′ ends of the targeting vector invade the chromosome in directions facing each other. In the chromosomal DSB-induced ends-in recombination, the broken chromosomal ends (jagged blue ovals) invade/anneal to the targeting vector in directions facing each other. (TIF) [file pgen.1004251.s003.tif]

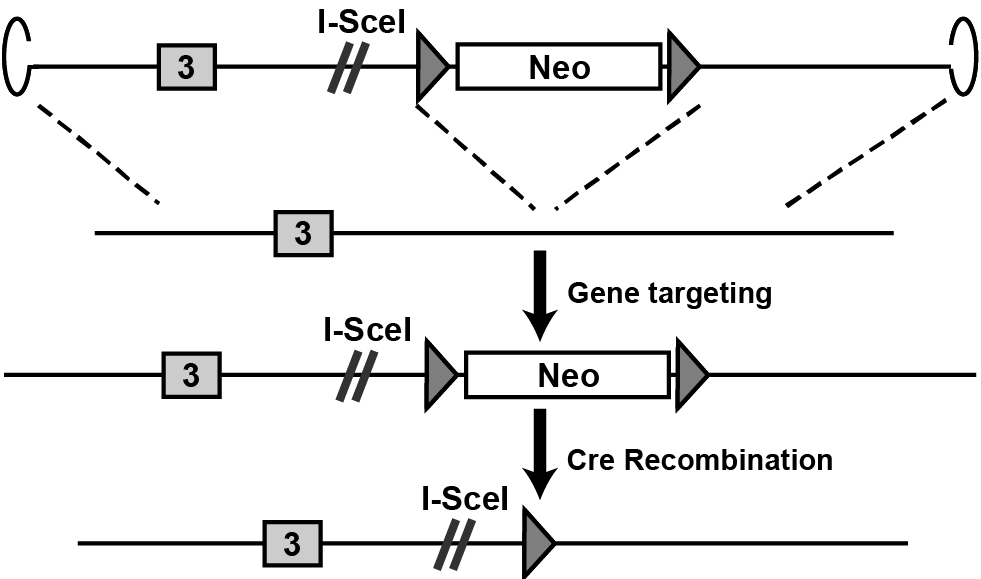

Supplement: Figure S4 — Construction of a human cell line containing an I-SceI site imbedded in the HPRT locus. A cartoon of a rAAV I-SceI knock-in targeting vector is shown on the top line. The I-SceI recognition site is shown a double-hatched line. A cartoon of the relevant portion of the HPRT locus (horizontal line with a rectangular exon 3) is shown on the line below. Following correct GTing, both the I-SceI recognition site and the NEO drug resistance gene are integrated at the HPRT locus. Following Cre recombination, the NEO gene is removed and a solo LoxP scar (shaded triangle) remains. (TIF) [file pgen.1004251.s004.tif]
