# Supplementary material for: The Mechanism of Gene Targeting in Human Somatic Cells
Source: PLoS Genet. 2014 Apr 3;10(4):e1004251. doi: 10.1371/journal.pgen.1004251 (PMC3974634; doi:10.1371/journal.pgen.1004251)
Supplement: Table S1 — SNP retention of rAAV gene targeting colonies in parental HCT116 cells. (PDF) [file pgen.1004251.s005.pdf]

S1. SNP retention of rAAV gene targeting colonies in parental HCT116 cells.

Legends: Viral | Genomic

NdeI	EcoRI	LHP	NcoI	AseI	SspI	SacI	RHP	XbaI	SbfI	#	%
+	+	+	+	+	+	+	-	-	-	5	2.2%
+	+	+	+	+	+	-	-	-	-	1	0.4%
+	+	+	+	+	-	+	-	-	+	1	0.4%
-	+	+	+	+	+	+	+	+	-	2	0.9%
-	+	+	+	+	+	+	+	-	-	14	6.1%
-	+	+	+	+	+	+	-	+	-	1	0.4%
-	+	+	+	+	+	+	-	-	-	9	3.9%
-	+	+	+	+	+	-	-	-	+	2	0.9%
-	+	+	+	+	+	-	-	-	-	17	7.4%
-	+	+	+	+	-	+	-	-	-	1	0.4%
-	+	+	+	+	-	-	-	-	-	7	3.0%
-	+	+	-	+	+	-	-	-	-	2	0.9%
-	+	-	+	+	+	+	-	-	-	2	0.9%
-	+	-	+	+	-	-	-	-	-	1	0.4%
-	+	-	-	-	+	+	+	+	-	1	0.4%
-	-	+	+	+	+	+	+	+	-	3	1.3%
-	-	+	+	+	+	+	+	-	-	3	1.3%
-	-	+	+	+	+	+	-	-	-	6	2.6%
-	-	+	+	+	+	-	+	-	-	2	0.9%
-	-	+	+	+	+	-	-	-	-	13	5.7%
-	-	+	+	+	-	-	-	-	-	4	1.7%
-	-	+	-	+	+	+	+	+	-	11	4.8%
-	-	+	-	+	+	+	+	-	-	5	2.2%
-	-	+	-	+	+	-	+	+	-	2	0.9%
-	-	+	-	+	+	-	-	-	-	1	0.4%
-	-	+	-	+	-	+	+	-	-	1	0.4%
-	-	+	-	+	-	-	+	-	-	1	0.4%
-	-	+	-	-	-	+	+	+	-	1	0.4%
-	-	-	+	+	+	+	+	+	+	2	0.9%
-	-	-	+	+	+	+	+	+	-	3	1.3%
-	-	-	+	+	+	+	+	-	-	1	0.4%
-	-	-	+	+	+	+	-	-	-	3	1.3%
-	-	-	+	+	+	-	+	-	-	1	0.4%
-	-	-	+	+	+	-	-	-	-	1	0.4%
-	-	-	-	+	+	+	+	+	+	5	2.2%
-	-	-	-	+	+	+	+	+	-	35	15.2%
-	-	-	-	+	+	+	+	-	-	16	7.0%
-	-	-	-	+	+	+	-	+	+	3	1.3%
-	-	-	-	+	+	+	-	+	-	2	0.9%
-	-	-	-	+	+	+	-	-	-	6	2.6%
-	-	-	-	+	+	-	+	-	-	2	0.9%
-	-	-	-	+	+	-	-	-	-	19	8.3%
-	-	-	-	+	-	+	+	+	-	2	0.9%
-	-	-	-	+	-	-	-	-	-	2	0.9%
-	-	-	-	-	+	+	+	+	-	3	1.3%
-	-	-	-	-	+	+	-	-	-	1	0.4%
-	-	-	-	-	+	-	-	-	-	4	1.7%
**7**	**66**	**115**	**105**	**220**	**209**	**148**	**116**	**76**	**13**	**230**	**100.00%**
**3.04%**	**28.70%**	**50.00%**	**45.65%**	**95.65%**	**90.87%**	**64.35%**	**50.43%**	**33.04%**	**5.65%**		
