# Supplementary material for: The Mechanism of Gene Targeting in Human Somatic Cells
Source: PLoS Genet. 2014 Apr 3;10(4):e1004251. doi: 10.1371/journal.pgen.1004251 (PMC3974634; doi:10.1371/journal.pgen.1004251)
Supplement: Table S2 — SNP retention of rAAV random integration colonies in parental HCT116 cells. (PDF) [file pgen.1004251.s006.pdf]

S2. SNP retention of rAAV random integration colonies in parental HCT116 cells.

**Legends:**	Viral	Genomic

NdeI	EcoRI	LHP	NcoI	AseI	SspI	SacI	RHP	XbaI	SbfI
+	+	+	+	+	+	+	+	+	+
+	+	+	+	+	+	+	+	+	+
+	+	+	+	+	+	+	+	+	+
+	+	+	+	+	+	+	+	+	+
+	+	+	+	+	+	+	+	+	+
+	+	+	+	+	+	+	+	+	+
+	+	+	+	+	+	+	+	+	+
+	+	+	+	+	+	+	+	+	+
+	+	+	+	+	+	+	+	+	+
+	+	+	+	+	+	+	+	+	+
+	+	+	+	+	+	+	+	+	+
+	+	+	+	+	+	+	+	+	+
+	+	+	+	+	+	+	+	+	+
+	+	+	+	+	+	+	+	+	+
+	+	+	+	+	+	+	+	+	+
+	+	+	+	+	+	+	+	+	+
+	+	+	+	+	+	+	+	+	+
+	+	+	+	+	+	+	+	+	+
+	+	+	+	+	+	+	+	+	+
+	+	+	+	+	+	+	+	+	+
+	+	+	+	+	+	+	+	+	+
+	+	+	+	+	+	+	+	+	+
+	+	+	+	+	+	+	+	+	+
+	+	+	+	+	+	+	+	+	+
+	+	+	+	+	+	+	+	+	+
+	+	+	+	+	+	+	+	+	+
+	+	+	+	+	+	+	+	+	+
+	+	+	+	+	+	+	+	+	+
+	+	+	+	+	+	+	+	+	+
+	+	+	+	+	+	+	+	+	+
+	+	+	+	+	+	+	+	+	+
+	+	+	+	+	+	+	+	+	+
+	+	+	+	+	+	+	+	+	+
+	+	+	+	+	+	+	+	+	+
+	+	+	+	+	+	+	+	+	+
+	+	+	+	+	+	+	+	+	+
+	+	+	+	+	+	+	+	+	+
+	+	+	+	+	+	+	+	+	+
**37**	**37**	**37**	**37**	**37**	**37**	**37**	**37**	**37**	**37**
**100.00%**	**100.00%**	**100.00%**	**100.00%**	**100.00%**	**100.00%**	**100.00%**	**100.00%**	**100.00%**	**100.00%**
