# Supplementary material for: The Mechanism of Gene Targeting in Human Somatic Cells
Source: PLoS Genet. 2014 Apr 3;10(4):e1004251. doi: 10.1371/journal.pgen.1004251 (PMC3974634; doi:10.1371/journal.pgen.1004251)
Supplement: Table S3 — SNP retention of plasmid-based gene targeting in parental HCT116 cells. (PDF) [file pgen.1004251.s007.pdf]

S3. SNP retention of plasmid-based gene targeting in parental HCT116 cells.

							**Legends:**	Viral	Genomic
**NdeI**	**EcoRI**	**LHP**	**NcoI**	**AseI**	**SspI**	**SacI**	**RHP**	**XbaI**	**SbfI**
+	+	+	+	+	+	-	-	-	-
-	+	+	+	+	+	+	-	+	-
-	+	+	+	+	+	+	-	+	-
-	+	+	+	+	+	-	-	-	-
-	+	-	-	+	+	+	-	-	-
-	-	+	+	+	+	+	+	+	+
-	-	+	+	+	+	+	+	+	-
-	-	+	+	+	+	+	-	-	-
-	-	+	+	+	+	-	-	-	-
-	-	+	+	+	+	-	-	-	-
-	-	+	+	+	-	+	-	-	-
-	-	-	+	+	+	+	+	-	-
-	-	-	-	+	+	+	+	+	+
-	-	-	-	+	+	+	+	-	-
-	-	-	-	+	+	+	-	-	-
-	-	-	-	-	+	+	+	+	-
-	-	-	-	-	+	+	-	+	-
-	-	-	-	-	-	-	-	-	-
**1**	**5**	**10**	**11**	**15**	**16**	**13**	**6**	**7**	**2**
**5.56%**	**27.78%**	**55.56%**	**61.11%**	**83.33%**	**88.89%**	**72.22%**	**33.33%**	**38.89%**	**11.11%**
