# Supplementary material for: The Mechanism of Gene Targeting in Human Somatic Cells
Source: PLoS Genet. 2014 Apr 3;10(4):e1004251. doi: 10.1371/journal.pgen.1004251 (PMC3974634; doi:10.1371/journal.pgen.1004251)
Supplement: Table S5 — Subcloning of colonies from Table S4 that are sectored on both arms. (PDF) [file pgen.1004251.s009.pdf]

S5. Subcloning of colonies from Supplemental Table S4 that are sectored on both arms.

											Legends:	Viral	Genomic	Unsectored
**Clone**	**Subclone**	**NdeI**	**EcoRI**	**LHP**	**NcoI**	**AseI**	**SspI**	**SacI**	**RHP**	**XbaI**	**SbfI**	***Trans***	***Cis***	**Noncanonical**
#1	1	-	-	-	-	+	+	+	+	+	-	1		
	2	+	+	+	+	+	+	+	-	-	-	1		
	3	+	+	+	+	+	+	+	-	-	-	1		
	4	-	-	-	-	+	+	+	+	+	-	1		
	5	+	+	+	+	+	+	+	-	-	-	1		
	6	+	+	+	+	+	+	+	-	-	-	1		
	7	+	+	+	+	+	+	+	-	-	-	1		
	8	-	-	-	-	+	+	+	+	+	-	1		
#2	1	+	+	+	+	+	+	+	-	-	-	1		
	2	-	-	-	+	+	+	+	+	+	-	1		
	3	-	-	-	+	+	+	+	+	+	-	1		
	4	-	-	-	+	+	+	+	+	+	-	1		
	5	-	-	-	+	+	+	+	+	+	-	1		
	6	+	+	+	+	+	+	+	-	-	-	1		
	7	+	+	+	+	+	+	+	-	-	-	1		
	8	+	+	+	+	+	+	+	-	-	-	1		
#3	1	-	-	+	-	+	+	+	+	+	+	1		
	2	+	+	+	+	+	+	-	-	-	-	1		
	3	-	-	+	-	+	+	+	+	+	+	1		
	4	-	-	+	-	+	+	+	+	+	+	1		
#4	1	-	-	-	-	+	+	+	+	+	-	1		
	2	-	+	+	+	+	+	+	+	-	-	1		
	3	-	+	+	+	+	+	+	+	-	-	1		
	4	-	-	-	-	+	+	+	+	+	-	1		
	5	-	-	-	-	+	+	+	+	+	-	1		
	6	-	-	-	-	+	+	+	+	+	-	1		
	7	-	-	-	-	+	+	+	+	+	-	1		
#5	1	-	-	-	-	+	+	+	+	+	-	1		
	2	-	+	+	+	+	+	+	+	-	-	1		
	3	-	-	-	-	+	+	+	+	+	-	1		
	4	-	+	+	+	+	+	+	+	+	-			1
	5	-	-	-	-	+	+	+	+	+	-	1		
	6	-	+	+	+	+	+	+	+	-	-	1		
	7	-	-	-	-	+	+	+	+	+	-	1		
	8	-	+	+	+	+	+	+	+	-	-	1		
#6	1	-	+	+	+	+	+	+	-	-	-	1		
	2	-	-	-	-	+	+	+	-	-	-		1	
	3	-	-	-	-	+	+	+	+	+	-	1		
	4	-	+	+	+	+	+	+	-	-	-	1		
	5	-	+	+	+	+	+	+	+	+	-		1	
	6	-	+	+	+	+	+	+	-	-	-	1		
	7	-	+	+	+	+	+	+	-	-	-	1		
	8	-	-	-	-	+	+	+	+	+	-	1		
#7	1	-	-	-	-	+	+	-	-	-	-		1	
	2	-	-	-	-	+	+	+	+	+	-	1		
	3	-	+	+	+	+	+	-	-	-	-	1		
	4	-	+	+	+	+	+	-	-	-	-	1		
	5	-	+	+	+	+	+	-	-	-	-	1		
	6	-	+	+	+	+	+	+	+	+	-		1	
#8	1	-	+	+	+	+	+	-	-	-	-	1		
	2	-	-	-	-	+	+	+	-	-	-	1		
	3	-	+	+	+	+	+	-	-	-	-	1		
	4	-	+	+	+	+	+	-	-	-	-	1		
	5	-	+	+	+	+	+	-	-	-	-	1		
	6	-	-	-	-	+	+	+	-	-	-	1		
	7	-	+	+	+	+	+	-	-	-	-	1		
	8	-	-	-	-	+	+	+	-	-	-	1		
#9	1	-	+	+	+	+	-	-	-	-	-	1		
	2	-	+	+	+	+	-	-	-	-	-	1		
	3	-	-	-	-	+	+	+	+	+	-	1		
	4	-	+	+	+	+	+	+	+	-	-			1
#10	1	-	-	-	-	+	+	+	+	+	-	1		
	2	-	-	-	+	+	+	-	-	-	-	1		
	3	-	-	-	+	+	+	-	-	-	-	1		
	4	-	-	-	-	+	+	+	+	+	-	1		
	5	-	-	-	-	+	+	+	+	+	-	1		
	6	-	-	-	-	+	+	+	+	+	-	1		
	7	-	-	-	-	+	+	+	+	+	-	1		
	8	-	-	-	-	+	+	+	+	+	-	1		
#11	1	-	+	+	+	+	+	-	-	-	-	1		
	2	-	+	+	+	+	+	-	-	-	-	1		
	3	-	+	+	+	+	+	-	-	-	-	1		
	4	-	-	-	-	-	+	+	+	+	-	1		
	5	-	-	-	-	-	+	+	+	+	-	1		
	6	-	-	-	-	-	+	-	-	-	-		1	
	7	-	-	-	-	-	+	-	-	-	-		1	
	8	-	+	+	+	+	+	-	-	-	-	1		
Note: Colonies generated by two independent GTing events have an equal chance to become *trans* or *cis*												89.61%	7.79%	2.82%

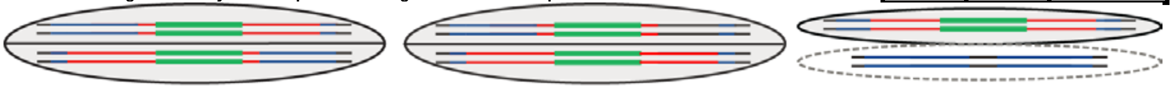

Trans*: canonical HR**   ***Cis*: two independent GTing, *etc.   **Unsectored: SSA**
