# Supplementary material for: The Mechanism of Gene Targeting in Human Somatic Cells
Source: PLoS Genet. 2014 Apr 3;10(4):e1004251. doi: 10.1371/journal.pgen.1004251 (PMC3974634; doi:10.1371/journal.pgen.1004251)
Supplement: Table S6 — The sectoring assay in parental DLD-1 cells. (PDF) [file pgen.1004251.s010.pdf]

S6. The sectoring assay in parental DLD-1 cells.

Legends: Viral | Sectored | Genomic

NdeI	EcoRI	LHP	NcoI	AseI	SspI	SacI	RHP	XbaI	SbfI	Sectored
-	+/-	+	+	+	+	+/-	+	+	-	2
-	+/-	+	+	+	+	+/-	+	-	-	2
-	+/-	+	+	+	+	+/-	+/-	+/-	-	2
-	+/-	+	+	+	-	+/-	+/-	+/-	-	2
-	+/-	+	+/-	+	+	+	+	+/-	-	2
-	+/-	+	+/-	+	+	+	+/-	-	-	2
-	+/-	+	+/-	+	+	+/-	+	+	-	2
-	+/-	+	+/-	+	+	+/-	+/-	+/-	+/-	2
-	+/-	+	+/-	+	+	+/-	-	+	-	2
-	+/-	+	+/-	+	+	+/-	-	-	-	2
-	+/-	+	+/-	+	+	-	+	+/-	-	2
-	+/-	+/-	+/-	+	+	+/-	+/-	+	-	2
-	+/-	+/-	+/-	+	+	+/-	-	+/-	-	2
-	+/-	+/-	+/-	+	+	+/-	-	-	-	2
-	+/-	+/-	+/-	+	+	+/-	-	-	-	2
-	+/-	+/-	+/-	+	-	+/-	+/-	-	-	2
-	+/-	-	+	+	+	+/-	-	+	-	2
-	+/-	-	+/-	+	+	+/-	+	+	-	2
-	+/-	-	+/-	+	+	+/-	-	-	-	2
-	+/-	-	+/-	+	+/-	+	+	+	-	2
-	-	+/-	+/-	+	+/-	+/-	-	-	-	2
-	-	-	+/-	+	+	+	+	+/-	-	2
-	-	-	+/-	+	+	+	+	+/-	-	2
-	-	-	+/-	+	+	+/-	+	+/-	-	2
-	-	-	+/-	+	+	+/-	+/-	+/-	-	2
-	-	-	+/-	+	+	+/-	-	-	-	2
+/-	+	-	+	+	+	+	+	+	+	1
+/-	+/-	+	+	+	+	+	+	-	-	1
-	+/-	+	+/-	+	+	-	+	-	-	1
-	+/-	+/-	+/-	+	+	+	+	-	-	1
-	+/-	+/-	+/-	+	+	-	-	-	-	1
-	+/-	+/-	+/-	+	+	-	-	-	-	1
-	-	+/-	+/-	+	+	-	-	-	-	1
-	-	-	+/-	-	-	-	-	-	-	1
+	+	+	+	+	+	+	+	+/-	-	1
+	+	+	+	+	-	+/-	+	+/-	-	1
-	+	+	+	+	+	+/-	-	-	+/-	1
-	+	+	+	+	+	+/-	-	-	-	1
-	-	+	+	+	+	+/-	+/-	-	-	1
-	-	+	+	+	+	+/-	-	-	-	1
-	-	-	-	+	+	+/-	-	-	-	1
-	+	+	+	+	+	+	-	-	-	0
-	-	+	+	+	+	+	-	-	-	0
-	-	+	-	+	+	+	+	-	-	0
-	-	-	-	+	+	+	+	+	-	0
-	-	-	-	-	+	+	-	-	-	0
**2**	**25**	**10***	**27**	**0**	**2**	**25**	**8**	**12**	**2**	**41**
**4.35%**	**54.35%**	**21.74%**	**58.70%**	**0.00%**	**4.35%**	**54.35%**	**17.39%**	**26.09%**	**4.35%**	**89.13%**

Note: The hairpins are composed of 3-4 clustered mismatches. In the LHP, the genomic sequence CcagcTAcG basepairs with TcagcCTcC in the virus, whereas in RHP, TAC basepairs with ATG. These hairpins are sectored at a significantly lower frequency compared to the flanking SNPs in DLD-1 but not HCT116 cell line. Also in DLD-1 , the TAcG/CTcC mismatch cluster in the LHP is sectored in only 3 clones, compared to the singular T/C mismatch which is sectored in 10 clones*. These results indicate that the DLD-1 cell line can resolve clustered mismatches that can form short mismatch loops via the MSH2/MSH3 pathway, whereas the HCT116 cell line is deficient in both single
