# Supplementary material for: The Mechanism of Gene Targeting in Human Somatic Cells
Source: PLoS Genet. 2014 Apr 3;10(4):e1004251. doi: 10.1371/journal.pgen.1004251 (PMC3974634; doi:10.1371/journal.pgen.1004251)
Supplement: Table S8 — SNP retention of I-SceI-induced rAAV gene targeting on the left homology arm. (PDF) [file pgen.1004251.s012.pdf]

S8. SNP retention of *I-SceI* -induced rAAV gene targeting on the left homology arm.

Legends: Viral (shaded) | Genomic (unshaded)

NdeI	EcoRI	LHP	NcoI	AseI	SspI	SacI	RHP	XbaI	SbfI
-	-	+	+	+	+	+	ND	+	+
-	-	+	+	+	+	-	ND	-	-
-	+	+	+	+	+	+	ND	+	-
-	-	+	+	+	+	+	ND	-	-
-	-	-	+	+	+	+	ND	+	+
-	-	-	+	+	+	+	ND	+	-
-	-	+	+	+	+	-	ND	-	-
-	-	-	+	+	+	+	ND	-	-
-	+	+	+	+	+	+	ND	-	-
-	+	+	+	+	+	+	ND	-	-
-	+	+	+	+	+	+	ND	+	-
-	+	+	+	+	+	+	ND	-	-
+	+	+	+	+	+	+	ND	-	-
-	-	+	+	+	+	+	ND	+	+
-	-	-	+	+	+	-	ND	-	-
-	+	+	+	+	+	-	ND	-	-
-	-	-	+	+	+	+	ND	+	+
-	-	-	+	+	+	+	ND	+	-
+	+	+	+	+	+	+	ND	+	+
-	+	+	+	+	+	+	ND	+	-
-	-	-	+	+	+	+	ND	+	-
-	-	-	+	+	+	+	ND	-	-
-	-	+	+	+	+	+	ND	-	-
-	+	+	+	+	+	-	ND	-	-
-	-	-	+	+	+	+	ND	+	-
-	+	+	+	+	+	+	ND	+	-
-	+	+	+	+	+	+	ND	-	-
-	+	+	+	+	+	-	ND	-	-
-	-	-	+	+	+	+	ND	-	-
-	+	+	+	+	+	+	ND	+	-
-	+	+	+	+	+	+	ND	+	-
-	+	+	+	+	-	-	ND	-	-
-	-	+	+	+	+	-	ND	-	-
-	-	+	+	+	+	-	ND	+	-
+	+	+	+	+	+	+	ND	-	-
-	-	+	+	+	+	+	ND	+	-
-	-	+	+	+	+	+	ND	-	-
-	-	-	+	+	+	+	ND	+	-
-	-	+	+	+	-	-	ND	-	-
-	-	+	+	+	+	+	ND	-	-
+	+	+	+	+	+	+	ND	-	-
-	-	+	+	+	-	-	ND	-	-
-	-	+	+	+	+	+	ND	+	-
+	-	+	+	+	+	-	ND	-	-
+	+	+	+	+	+	+	ND	-	-
-	-	-	+	+	+	+	ND	+	-
-	-	-	+	+	+	+	ND	-	-
-	-	+	+	+	+	+	ND	-	-
**6**	**19**	**35**	**48**	**48**	**45**	**36**	**ND**	**20**	**5**
**12.50%**	**39.58%**	**72.92%**	**100.00%**	**100.00%**	**93.75%**	**75.00%**	**ND**	**41.67%**	**10.42%**
