# Supplementary material for: The Mechanism of Gene Targeting in Human Somatic Cells
Source: PLoS Genet. 2014 Apr 3;10(4):e1004251. doi: 10.1371/journal.pgen.1004251 (PMC3974634; doi:10.1371/journal.pgen.1004251)
Supplement: Table S9 — SNP retention of rAAV gene targeting colonies in MLH1+ HCT116 cells. (PDF) [file pgen.1004251.s013.pdf]

S9. SNP retention of rAAV gene targeting colonies in *MLH1*$^{+}$ HCT116 cells.

Legends: Viral | Genomic

NdeI	EcoRI	LHP	NcoI	AseI	SspI	SacI	RHP	XbaI	SbfI
+	+	+	+	+	+	+	-	-	-
-	+	+	+	+	+	+	+	+	-
-	+	+	+	+	+	+	+	-	-
-	+	+	+	+	+	+	+	-	-
-	+	+	+	+	+	-	-	-	-
-	-	+	+	+	+	+	+	-	-
-	-	+	+	+	+	+	-	+	-
-	-	+	+	+	+	-	+	-	-
-	-	+	+	+	+	-	-	-	-
-	-	+	+	+	-	-	+	-	-
-	-	-	+	+	+	+	-	-	-
-	-	-	-	+	+	+	+	+	-
-	-	-	-	+	+	+	+	+	-
-	-	-	-	+	+	+	+	+	-
-	-	-	-	+	+	+	-	+	-
-	-	-	-	+	+	-	-	-	-
-	-	-	-	+	+	-	-	-	-
-	-	-	-	+	+	-	-	-	-
-	-	-	-	+	-	+	-	-	-
-	-	-	-	+	-	-	-	-	-
**1**	**5**	**10**	**11**	**20**	**17**	**12**	**9**	**6**	**0**
**5.00%**	**25.00%**	**50.00%**	**55.00%**	**100.00%**	**85.00%**	**60.00%**	**45.00%**	**30.00%**	**0.00%**
